# The Interaction Among Rhizosphere Soil Nutrients, Metabolites, and Microbes Determines the Productivity of Perennial Cultivated Grassland in Qinghai‐Tibet Plateau

**DOI:** 10.1002/ece3.71149

**Published:** 2025-04-23

**Authors:** Xiaofang Zhang, Yuzhen Liu, Quan Cao, Zengzeng Yang, Zehang Yu, Caidi Li, Chunping Zhang, Quanmin Dong

**Affiliations:** ^1^ Academy of Animal Science and Veterinary Medicine Qinghai University Xining Qinghai China; ^2^ Qinghai Provincial Key Laboratory of Adaptive Management on Alpine Grassland Xining Qinghai China; ^3^ Key Laboratory of the Alpine Grassland Ecology in the Three Rivers Region Qinghai University, Ministry of Education Xining Qinghai China

**Keywords:** bacterial community structure, perennial cultivated grassland, productivity, Qinghai‐Tibet plateau, rhizosphere metabolites

## Abstract

The rhizosphere, as the primary site of plant–soil –microbe interactions, plays an important role in plant productivity. The influence of plant species on rhizosphere soil properties and how this influence shapes the productivity of grassland ecosystems remains poorly understood. Therefore, this study analyzed the changes in bacterial community structure and metabolites in the rhizosphere soil of perennial cultivated grassland. The aim was to elucidate the pathways and underlying mechanisms by which soil physicochemical properties, bacterial community structure, and rhizosphere metabolites influence productivity. Taking perennial monocropping grasslands established with three common grass species from the Qinghai –Tibet Plateau as the research objects, a comprehensive study was conducted using replicated field trials. Rhizosphere soil samples were collected from *Elymus breviaristatus* , *Festuca sinensis*, and *Poa pratensis* . The results indicated significant differences in productivity, soil physicochemical properties, bacterial diversity, and community structure among the monocropping grasslands. Among them, ANPP (589.17 g·m^−2^), root biomass (3601.67 g·m^−2^), moisture (18.15%) and *Verrucomicrobiota* (3.60%) of *Elymus breviaristatus* are higher than those of *Festuca sinensis* and *Poa pratensis* , while the relative abundance of *Firmicutes* (0.82%) is lower than that of *Festuca sinensis* and *Poa pratensis* . The topological characteristics of soil bacterial networks varied among the different grass species. The abundances of metabolites consisting of phenylalanine, proline, raffinose, maltotriose, uridine, and 2‐methylbenzaldehyde differed among different treatments. Pathway analysis highlighted the upregulation of ABC transporters and pyrimidine metabolism pathways in *Elymus breviaristatus* compared to *Festuca sinensis* and *Poa pratensis* . Moreover, *Elymus breviaristatus* secreted more uridine, which prevents the recruitment of pathogenic bacteria (such as *Firmicutes*) and promotes the recruitment of *Verrucomicrobiota*, thus improving grassland productivity. These findings show that the productivity of perennial monocropping grasslands around Qinghai Lake is the result of the interaction among rhizosphere soil nutrients, metabolites, and microorganisms. From the ecological point of view, *Elymus breviaristatus* is more conducive to the improvement of forage yield and the restoration of degraded grassland.

## Graphical abstract

The productivity of monocropping grasslands is significantly correlated with soil physicochemical properties, root biomass, differential metabolites, and microbial contents. Random forest analysis further identified Uridine, Moisture, AOC, AP, NO3– N and SBD as key factors driving productivity changes in perennial monocropping grasslands. These findings show that the productivity of perennial monocropping grasslands around Qinghai Lake is the result of the interaction among rhizosphere soil nutrients, metabolites, and microorganisms.

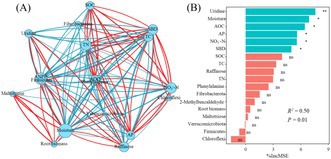

## 1 Introduction

The rhizosphere, as the primary site of plant–soil –microbe interactions, serves as a crucial bridge connecting plants and soil in ecosystems and is an active zone for nutrient cycling (Lv et al. 2024). Extensive literature has demonstrated the substantial potential of microorganisms in enhancing plant nutrition, yield, and tolerance to pathogens and abiotic stresses (Zhao, Zhang, et al. 2022). A comprehensive understanding of grassland productivity requires exploring its associated microbiota. The progress in DNA sequencing and biocomputing has opened avenues for exploring the genetic diversity within host‐associated microbial communities, particularly the uncultured components (Wani, Akhtar, Naqash, et al. 2022). Among the various methods used to characterize microbiota diversity, amplicon sequencing is the most widely used (Wani, Akhtar, Singh, et al. 2022). For bacteria, amplification sequencing studies typically focus on the small‐subunit ribosomal RNA (16S) locus, recognized for its taxonomic and phylogenetic information. 16S amplicon sequencing elucidates the association and scaling of microbial diversity under different environmental conditions by comparing 16 s sequences between samples (Wani, Rahayu, et al. 2024). With the rapid development of high‐throughput sequencing technology, metagenomics has emerged. It can directly study the total DNA of all microorganisms in the environment, without the need for pure culture of individual microorganisms, so as to fully and deeply reveal the diversity and functional potential of rhizosphere microbial communities. Metagenomics enhances soil health, ecosystem management, disease diagnosis, and biotechnology through the study of microbial interactions and diversity (Wani, Chopra et al. 2024). The low cost of 16S can be utilized to screen the target community after detecting the composition and diversity of a large number of samples in the early stage, and then conduct metagenomic studies to excavate the functional information of the community.

Rhizosphere microorganisms are vital for plant productivity and health. On one hand, the quantity and diversity of rhizosphere microorganisms influence plant growth, development, and health status. Beneficial microorganisms can promote plant growth and enhance plant stress resistance by increasing the availability and absorption of soil nutrients (Solomon et al. 2024). On the other hand, rhizosphere microorganisms drive nutrient cycling, such as carbon, nitrogen, and phosphorus, in the soil, significantly impacting plant health, nutrition, and productivity. They also regulate plant growth by producing various plant hormones or signaling molecules. Therefore, rhizosphere microbial communities can directly affect plant performance, either positively or negatively (Yan et al. 2022). The soil microenvironment, including soil physical and chemical properties, microbial populations, trace element content, and other indicators, influences plant nutrient absorption by regulating factors such as soil moisture, fertility, and physical, chemical, and biological processes. This, in turn, enhances plant growth and development, achieving high crop yields (Arvidsson 1999). Studies have found that soil environmental factors significantly impact microbial community structure, with root metabolites playing a crucial regulatory role in this process (Liu et al. 2022). This indicates a close relationship between the soil environment and soil microorganisms. Research has revealed a co‐evolutionary relationship between plants and soil microorganisms (Zhao et al. 2021). Plants actively secrete compounds that drive and shape microbial selection. These compounds specifically stimulate or inhibit different microbial members of the soil community, thereby shaping the rhizosphere microbiome (Wang et al. 2023). This interaction optimizes the nutritional benefits provided by plant‐associated microorganisms and promotes plant growth (Bakker et al. 2012). However, current research on the rhizosphere primarily focuses on forestry and agriculture, with limited studies on rhizosphere processes in cultivated grasslands.

Metabolites, which are essential products or intermediate compounds produced through enzymatic reactions, are critical for regulating interactions between soil rhizosphere microbial communities and plant populations (Zhao, Yao et al. 2022). These interactions ultimately impact plant growth and adaptability, particularly in the case of bioactive secondary metabolites (Zhuang et al. 2024). The compounds released by plant roots are diverse and include both primary and secondary metabolites. Typically, the secretion of primary metabolites exceeds that of secondary metabolites (Koprivova and Kopriva 2022). Primary metabolites are mainly substances that promote plant growth and development under stress conditions, while secondary metabolites can indirectly enhance plant adaptability by altering rhizosphere soil nutrients and microbial communities (Vives‐Peris et al. 2020). Recent studies have highlighted the high sensitivity of metabolites to changes in ecosystem productivity. For example, sugars and their derivatives, organic acids, phenols, and other secondary metabolites can regulate soil biochemical processes, leading to changes in rhizosphere soil metabolites (Haichar et al. 2014). The dynamic changes in the composition and relative abundance of these metabolites profoundly affect the physicochemical properties of rhizosphere soil, which in turn influence plant growth and development by modulating the absorption and transport of heavy metals in the soil (Jiang et al. 2024; Li et al. 2022). Additionally, modifications in soil metabolites induced by plant roots may also promote the colonization of beneficial microbial populations, resulting in various effects on plants (Su et al. 2023). The interaction between rhizosphere microbial communities and plant roots directly affects the maintenance of steady‐state conditions in the soil, thereby impacting plant health and yield. This interaction is mediated through dynamic changes in rhizosphere metabolites (She et al. 2021). Therefore, the study of soil microorganisms and plant interactions should not be limited to large and complex changes in microbial populations but should also include metabolites in the rhizosphere soil. However, research integrating microbial populations with metabolites in rhizosphere soil to explore similar response mechanisms remains limited (Liu et al. 2024).

The Qinghai Lake region is located in the cold high‐altitude farming and pastoral zone of the Tibetan Plateau, making this area ecologically fragile (Wang et al. 2020). Alpine grasslands are one of the most critical ecological function areas and represent a key geographic unit of this region. In recent years, significant degradation of the alpine grasslands has occurred due to both natural and anthropogenic factors, leading to relatively low grassland productivity and restricting the development of grassland‐based livestock farming (Zhang et al. 2019). To restore degraded grasslands, the government has implemented a series of measures, among which the establishment of cultivated grasslands is the most effective (Li et al. 2018). The establishment of cultivated grasslands is also a primary method for resolving the grass‐livestock conflict. Consequently, perennial cultivated grasslands, particularly monocropping cultivated grasslands, have rapidly developed in the region (Li et al. 2014). Studies by Zhao, Zhang, et al. (2022) indicate that interactions between bacterial genera are more complex in monocropping grasslands compared to other planting systems. Moreover, monocropping grasslands can enhance nitrogen fixation and productivity, and importantly, they strongly suppress soil‐borne pathogens, reducing soil‐transmitted diseases. Therefore, monocropping of gramineous species is a promising planting model for the Tibetan Plateau. At present, the native grasses commonly used in the cultivation of grassland in the alpine region are *Elymus breviaristatus* , *Festuca sinensis*, and *Poa pratensis* (Dong, Shang, et al. 2020). Research on monocropping cultivated grasslands in China mainly focuses on the effects of moisture, soil nutrients, harvest methods, and intensity on forage yield and quality. However, there are few studies analyzing the impact of different forage species around Qinghai Lake on rhizosphere soil microorganisms and metabolites, and their effects on productivity have rarely been reported (Tohtahun et al. 2024).

In this study, we used *Elymus breviaristatus* , *Festuca sinensis*, and *Poa pratensis* forage species commonly employed in the restoration of degraded grasslands on the Tibetan Plateau as test materials. Utilizing 16S gene sequencing and non‐targeted metabolomics, we systematically investigated the responses of rhizosphere soil nutrient content, metabolites, and the complex interactions among microbial communities in monocropping cultivated grasslands of different perennial grass genera. The aim of this study was to explore the variations in soil physicochemical properties, microbial communities, and rhizosphere metabolites across these three different monocropping grasslands, and to assess their impact on the productivity of grasslands in this ecologically significant region. Our hypotheses were: (1) There are differences in soil physicochemical properties, soil bacterial community, and metabolite composition among different species; (2) Interactions between rhizosphere metabolites and soil physicochemical properties affect the composition of the soil bacterial community, potentially enhancing the productivity of perennial monocropping grasslands.

## 2 Materials and Methods

### 2.1 Experimental Design and Sampling

Based on the local planting area, representative species from the genera *Elymus*, *Festuca*, and *Poa* were selected as experimental materials at Bakatai Farm and Pasture (101°5′E, 36°15′N) in Qinghai Province, China, at an average altitude of 3300 m. The region experiences a plateau continental climate with distinct cold and warm seasons and no absolute frost‐free period. The growing season is short and warm, while the dormant season is long and cold. Annual sunshine ranges from 2670.4 to 3036 h, with average temperatures between −5°C and 5°C. Precipitation averages about 338 mm per year, with over 75% falling during the warm season. The Dominant native vegetation includes species such as *Stipa purpurea*, *Kobresia capillifolia*, and *Potentilla nivea* . The soil type at the site is chestnut soil (Tong, Dong et al. 2024). The experimental plot was established in 2019, and the research objectives of this experiment were three monocropping treatments: *Elymus breviaristatus* monocropping, with an area of approximately 1.10 hm^2^ and a sowing density of 45.00 kg·hm^−2^; *Festuca sinensis* monocropping, with an area of approximately 1.10 hm^2^ and a sowing density of 30.00 kg·hm^−2^; and *Poa pratensis* monocropping, with an area of approximately 1.00 hm^2^ and a sowing density of 11.25 kg hm^−2^. The line spacing was 15 cm, and the sowing depth was 3–5 cm. Three replicates were performed per plot, and a completely random design was adopted. Urea and diammonium phosphate were mixed and applied at 75 kg hm^−2^ each. Urea topdressing was carried out in May every year, with a fertilization amount of 300 kg hm^−2^. The cultivated grassland was not grazed throughout the year.

### 2.2 Vegetation Biomass and Soil Sampling

During the growing season of pasture plants in August 2022, 10 uniformly growing plants were randomly selected from the experimental plots. A comprehensive root system was excavated to collect rhizosphere soil. Using the shaking technique (Chaudhary et al. 2015), soil that clung to plant roots after shaking was identified as rhizosphere soil (rhizosphere soil < 2 mm). After removing loose gravel and plant debris, the samples were processed in three ways: one portion was sieved through a 2 mm mesh and frozen at −80°C for microbial community and metabolite analysis; another was air‐dried for physicochemical testing; and the third was kept at 4°C for assessing soil ammonium nitrogen (NH_4_^+^‐N) and nitrate nitrogen (NO_3_^−^‐N) levels. After collecting the rhizosphere soil, the aboveground biomass and root biomass of the 10 sampled plants were obtained separately. Aboveground plant parts were placed in envelope bags, while root samples were collected in nylon mesh bags and thoroughly rinsed with water. Subsequently, the collected plant and root samples were brought back to the laboratory, placed in an oven at 105°C for 30 min, followed by further drying at 75°C until a constant dry weight was achieved to determine aboveground and belowground biomass. Representative samples from each experimental plot were obtained using random sampling methods. The subsamples, plant samples, and soil from each plot were thoroughly mixed and homogenized, resulting in 6 replicates for each treatment.

### 2.3 Soil Physical and Chemical Properties

Soil pH was determined by mixing soil with distilled water at a 1:5 ratio, shaking for 35 min, and measuring with a pH meter (Seven Excellence, Mettler‐Toledo, China) (Dong et al. 2023). Total carbon (TC) and total nitrogen (TN) were assessed using an elemental analyzer (Thermo Scientific FlashSmart, Thermo Fisher, Germany). Soil organic carbon (SOC) and active organic carbon (AOC) were measured via the K_2_Cr_2_O_7_ oxidation method with FeSO_4_ titration. Ammonium nitrogen (NH_4_^+^‐N) and nitrate nitrogen (NO_3_^−^‐N) were extracted using potassium chloride. Total phosphorus (TP) was evaluated with the molybdenum‐antimony colorimetric method, while available phosphorus (AP) was quantified through sodium bicarbonate extraction followed by molybdenum‐antimony colorimetry. The soil bulk density (SBD) and moisture content of the 0–10 cm soil layer were measured using a cutting ring (100 cm^3^) method (Huang et al. 2014).

### 2.4 Soil Bacterial Community Analysis

Total DNA from the microbial community was extracted using the cetyltrimethylammonium bromide (CTAB) method. The reagent, which was designed to uncover DNA from trace amounts of sample, has been shown to be effective for the preparation of DNA from most bacteria. Nuclear‐free water was used for the blank. The total DNA was eluted in 50 μL of Elution buffer and stored at −80°C until measurement in the PCR by LC‐Bio Technology Co. Ltd., Hang Zhou, Zhejiang Province, China. For amplification, the bacterial 16S rRNA gene's V3–V4 region was targeted using primers 341F (5′‐CCTACGGGNGGCWGCAG‐3′) and 805R (5′‐GACTACHVGGGTATCTAATCC‐3′). The 5′ ends of the primers were tagged with specific barcodes per sample and sequencing universal primers. PCR amplification was performed in a total volume of 25 μL reaction mixture containing 25 ng of template DNA, 12.5 μL PCR Premix, 2.5 μL of each primer, and PCR‐grade water to adjust the volume. The PCR conditions to amplify the prokaryotic 16S fragments consisted of an initial denaturation at 98°C for 30 s; 32 cycles of denaturation at 98°C for 10 s, annealing at 54°C for 30 s, and extension at 72°C for 45 s; and then final extension at 72°C for 10 min. The PCR products were confirmed with 2% agarose gel electrophoresis. Throughout the DNA extraction process, ultrapure water, instead of a sample solution, was used to exclude the possibility of false‐positive PCR results as a negative control. The PCR products were purified by AMPure XT beads (Beckman Coulter Genomics, Danvers, MA, USA) and quantified by Qubit (Invitrogen, USA). The amplicon pools were prepared for sequencing, and the size and quantity of the amplicon library were assessed on an Agilent 2100 Bioanalyzer (Agilent, USA) and with the Library Quantification Kit for Illumina (Kapa Biosciences, Woburn, MA, USA), respectively. The libraries were sequenced on the NovaSeq PE250 platform.

### 2.5 Rhizosphere Soil Metabolites

To prepare the samples, 100 mg of soil was weighed and ground in liquid nitrogen. Metabolites were then extracted with a 50% methanol solution. After adding 1 mL of chilled 50% methanol, the mixture was shaken at room temperature for 10 min and stored at −20°C overnight. Following centrifugation at 4000 *g* for 20 min, the supernatant was transferred to a new 96‐well plate for analysis. Samples not tested immediately were kept at −80°C until LC–MS analysis. Additionally, 10 μL from each sample was combined to form a QC sample. All samples underwent LC–MS non‐targeted metabolomics analysis. Firstly, all chromatographic separations were performed using a Vanquish Flex UHPLC system (Thermo Fisher Scientific, Bremen, Germany). An ACQUITY UPLC T3 column (100 × 2.1 mm, 1.8 μm, Waters, Milford, USA) was used for the reversed phase separation. The column oven was maintained at 35°C. The flow rate was 0.4 mL/min and the mobile phase consisted of solvent A (water, 0.1% formic acid) and solvent B (Acetonitrile, 0.1% formic acid). Gradient elution conditions were set as follows: 0 min to 0.5 min, 5% B; 0.5 min to 7 min, 5% to 100% B; 7 min to 8 min, 100% B; 8 min to 8.1 min, 100% to 5% B; 8.1 min to 10 min, 5% B. A high‐resolution tandem mass spectrometer Q‐Exactive (Thermo Scientific) was used to detect metabolites eluted from the column. The Q‐Exactive was operated in both positive and negative ion modes. Precursor spectra (70–1050 m/z) were collected at 70,000 resolution to hit an AGC target of 3e6. The maximum inject time was set to 100 ms. A top three configuration to acquire data was set in DDA mode. Fragment spectra were collected at 17,500 resolution to hit an AGC target of 1e5 with a maximum inject time of 80 ms. In order to evaluate the stability of the LC–MS during the whole acquisition, a quality control sample (Pool of all samples) was acquired after every 10 samples. The acquired MS data pretreatments, including peak picking, peak grouping, retention time correction, second peak grouping, and annotation of isotopes and adducts, were performed using XCMS software. LC–MS raw data files were converted into mzXML format and then processed by the XCMS, CAMERA, and metaX toolboxes implemented with the R software. The online KEGG, HMDB database was used to annotate the metabolites by matching the exact molecular mass data (m/z) of samples with those from thedatabase.

### 2.6 Statistical Analyses

One‐way ANOVA was conducted to assess the variations in biomass, soil physicochemical variables, relative abundance of diverse taxa, and α‐diversity indices among different monocropping grassland treatments (*p* < 0.05). Nonmetric multidimensional scaling (NMDS) and analysis of similarities (ANOSIM) were used to evaluate β‐diversity differences in microbial communities among treatments. Venn diagrams, generated using the “venn” package, illustrated shared and unique Operational Taxonomic Units (OTUs). Linear discriminant analysis effect size (LEfSe) was utilized to identify significant differences in microbial community composition among different monocropping treatments. Following normalization of OTU abundances and estimation of Spearman correlations between parameters, a Molecular Ecological Network (MEN) was constructed using the RMT model. Subsequently, Gephi 9.2 was used for network visualization and for the calculation of node, edge, and average degree. Differential metabolites were screened according to the Variable Importance in Projection (VIP), fold change (FC), and *P*‐value derived from the t‐test of the first principal component of the PLS‐DA model, with threshold values for significance of VIP > 1.0, FC ≥ 2, FC ≤ 1/2, and *p* < 0.05. KEGG is a database containing rich information on metabolic pathways and interactions. KEGG pathway enrichment analysis was used to identify the most significant signal transduction pathways and biological metabolic pathways related to the differential metabolites. Utilizing the analysis platform supplied by the sequencing company, we conducted PLS‐DA and KEGG pathway enrichment analyses of the rhizosphere metabolites. Furthermore, differential metabolites were identified using the Student's *t*‐test (*p* < 0.05). Pearson correlation analysis was conducted to explore relationships between rhizosphere soil nutrients, metabolites, microbial factors, and productivity. Random forest analysis was performed using the “randomForest” package. All statistical analyses were performed using R software (version 4.0.2).

## 3 Results

### 3.1 Vegetation Characteristics and Soil Physicochemical Properties

The vegetation characteristics (ANPP, Root biomass) and most soil physicochemical properties (TC, TN, SOC, AOC, NO_3_^−^‐N, AP, Moisture, SBD) of different monocropping grasslands showed significant differences (*p* < 0.05), while soil pH, NH_4_^+^‐N, and TP content did not show significant differences (Table 1). Among the different monocropping grasslands, the ANPP, root biomass, and Moisture were the highest in *Elymus breviaristatus* monocropping, while the soil chemical properties were the opposite, with the lowest total and available nutrient contents in *Elymus breviaristatus* monocropping.

**TABLE 1:** Changes in vegetation biomass and soil physicochemical properties of cultivated grassland under different treatments.

Variables	*Elymus breviaristatus*	*Festuca sinensis*	*Poa pratensis*
ANPP (g·m^−2^)	589.17 ± 28.86a	480.43 ± 15.23b	420.67 ± 9.05c
Root biomass (g·m^−2^)	3601.67 ± 417.01a	1962.91 ± 195.03b	2140.69 ± 394.46b
TC (g·kg^−1^)	32.64 ± 0.20b	34.67 ± 0.22a	34.03 ± 0.38a
TN (g·kg^−1^)	1.37 ± 0.03c	1.56 ± 0.05b	1.74 ± 0.08a
TP (g·kg^−1^)	0.72 ± 0.02	0.74 ± 0.01	0.76 ± 0.02
SOC (g·kg^−1^)	11.95 ± 0.15b	12.13 ± 0.16b	13.04 ± 0.24a
AOC (g·kg^−1^)	1.69 ± 0.02b	1.72 ± 0.01b	1.79 ± 0.02a
NH_4_^+^‐N (mg·kg^−1^)	22.94 ± 0.76	23.27 ± 0.51	23.73 ± 0.74
NO_3_^−^‐N (mg·kg^−1^)	13.23 ± 0.03c	13.38 ± 0.03b	13.67 ± 0.07a
AP (mg·kg^−1^)	2.38 ± 0.08b	2.44 ± 0.07b	2.74 ± 0.06a
pH	8.88 ± 0.04	8.80 ± 0.05	8.80 ± 0.06
Moisture (%)	18.15 ± 0.24a	12.46 ± 0.08b	11.68 ± 0.05c
SBD (g·cm^−3^)	1.18 ± 0.01c	1.23 ± 0.01b	1.33 ± 0.01a

*Note:* Lowercase letters indicate significant differences among different treatments (*p* < 0.05).

Abbreviations: ANPP, above‐ground net primary productivity; AOC, soil active organic carbon; AP, soil available phosphorus; NH_4_^+^‐N, soil ammonium nitrogen; NO_3_^−^‐N, soil nitrate‐nitrogen; SBD, soil bulk density; SOC, soil organic carbon; TC, soil total carbon; TN, soil total nitrogen; TP, soil total phosphorus.

### 3.2 Bacterial Diversity

**FIGURE 1:**
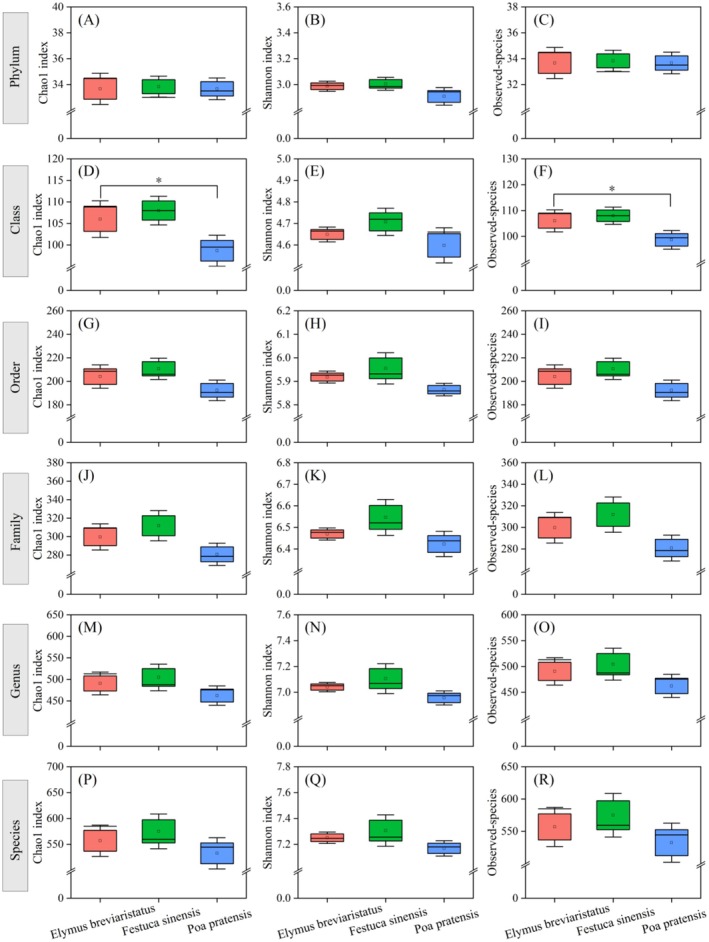
Changes of bacterial α‐diversity in cultivated grassland under different treatments. (A–C) represent the Chao1 index, Shannon index, and Observed‐species index, respectively, among different treatments at the phylum level; (D–F) represent the Chao1 index, Shannon index, and Observed‐species index, respectively, among different treatments at the class level; (G–I) represent the Chao1 index, Shannon index, and Observed‐species index, respectively, among different treatments at the order level; (J–L) represent the Chao1 index, Shannon index, and Observed‐species index, respectively, among different treatments at the family level; (M–O) represent the Chao1 index, Shannon index, and Observed‐species index, respectively, among different treatments at the genus level; (P–R) represent the Chao1 index, Shannon index, and Observed‐species index, respectively, among different treatments at the species level.

**FIGURE 2:**
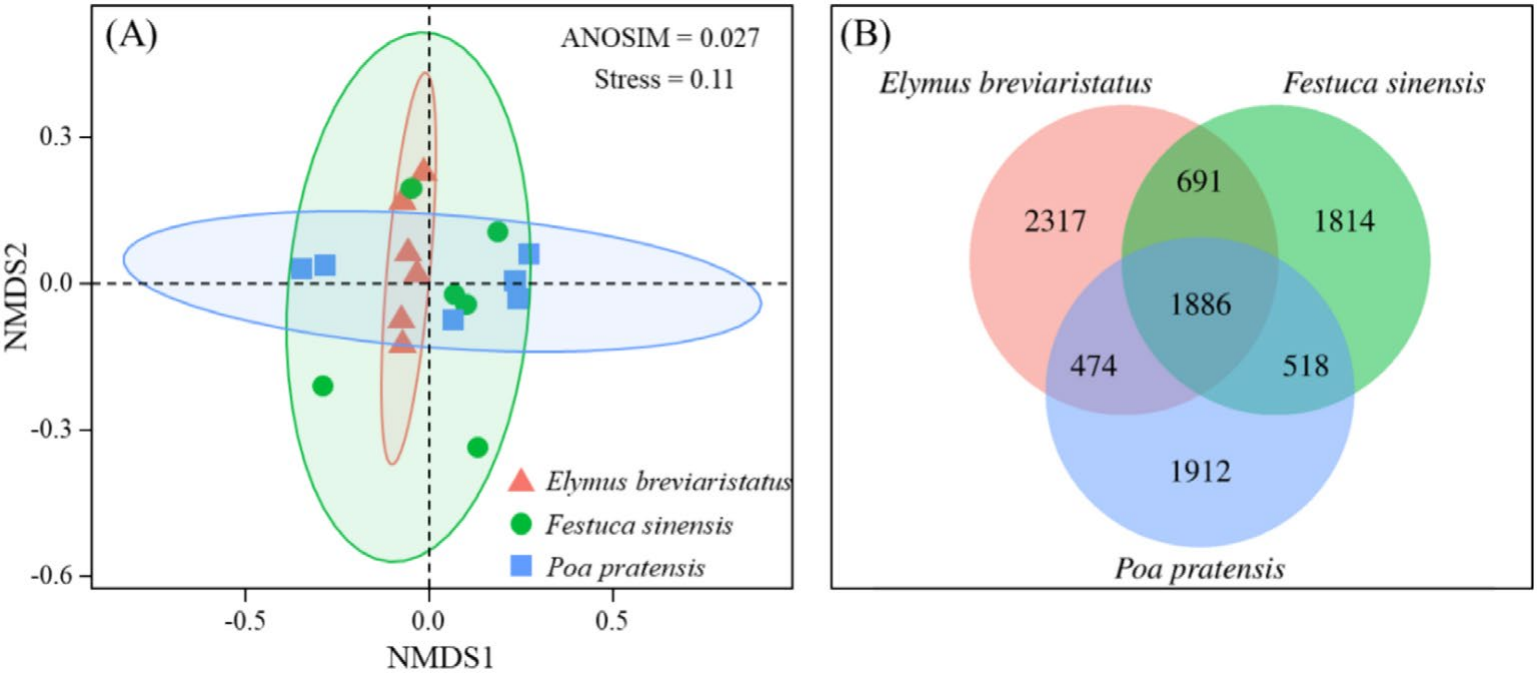
Based on the Bray distance of samples for the bacterial communities and Veen analysis, nonmetric multidimensional scaling (NMDS) ordination plot (A) and OTU (B) under different treatments.

The α‐diversity (Chao1, Shannon, Observed‐species) of bacteria phylum, class, order, family, genus, and species was analyzed. It was found that only the Chao1 and Observed‐species indices in different monocropping grasslands were significantly different at the class level (*p* < 0.05), while the others were not significantly different (*p* > 0.05). Among them, the α‐diversity of *Festuca sinensis* monocropping was significantly higher than that of *Elymus breviaristatus* and *Poa pratensis* monocropping; *Poa pratensis* monocropping had the lowest indicators (Figure 1). NMDS and ANOSIM tests based on Bray –Curtis distances indicated significant differences in bacterial community structures among different monocropping grasslands (Figure 2A).

### 3.3 Bacterial Community Composition

The numbers of OTUs for *Elymus breviaristatus* , *Festuca sinensis*, and *Poa pratensis* were 5368, 4909, and 4790, respectively. Among them, the number of OTUs shared by *Elymus breviaristatus* , *Festuca sinensis*, and *Poa pratensis* was 1886, accounting for 19.6% of the total OTUs. The number of unique OTUs for *Elymus breviaristatus* , *Festuca sinensis*, and *Poa pratensis* was 2317 (43.2%), 1814 (34%), and 1912 (39.9%), respectively (Figure 2B).

**FIGURE 3:**
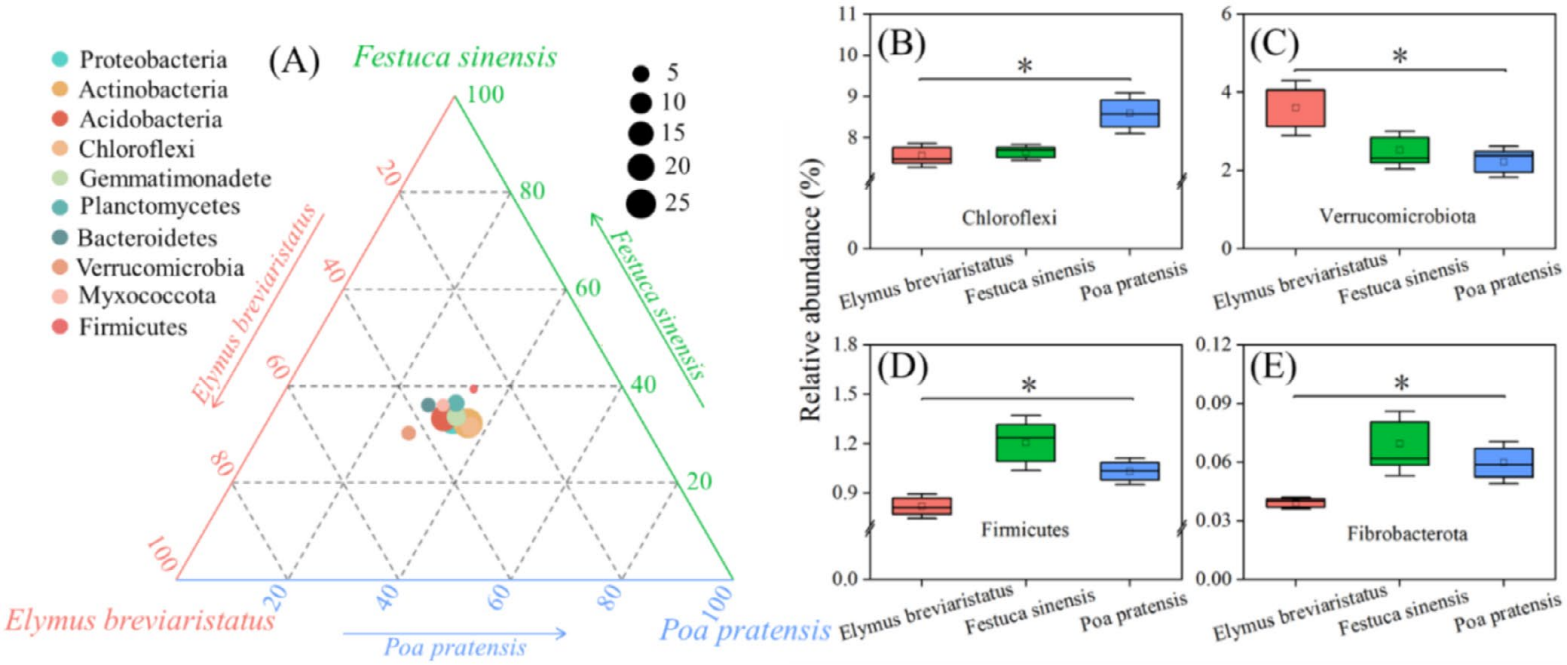
The relative abundance (%) of the bacterial community at the phylum level (A). The *Chloroflexi* (B), *Verrucomicrobiota* (C), *Firmicutes* (D) and *Fibrobacterota* (E) among different treatments.

**FIGURE 4:**
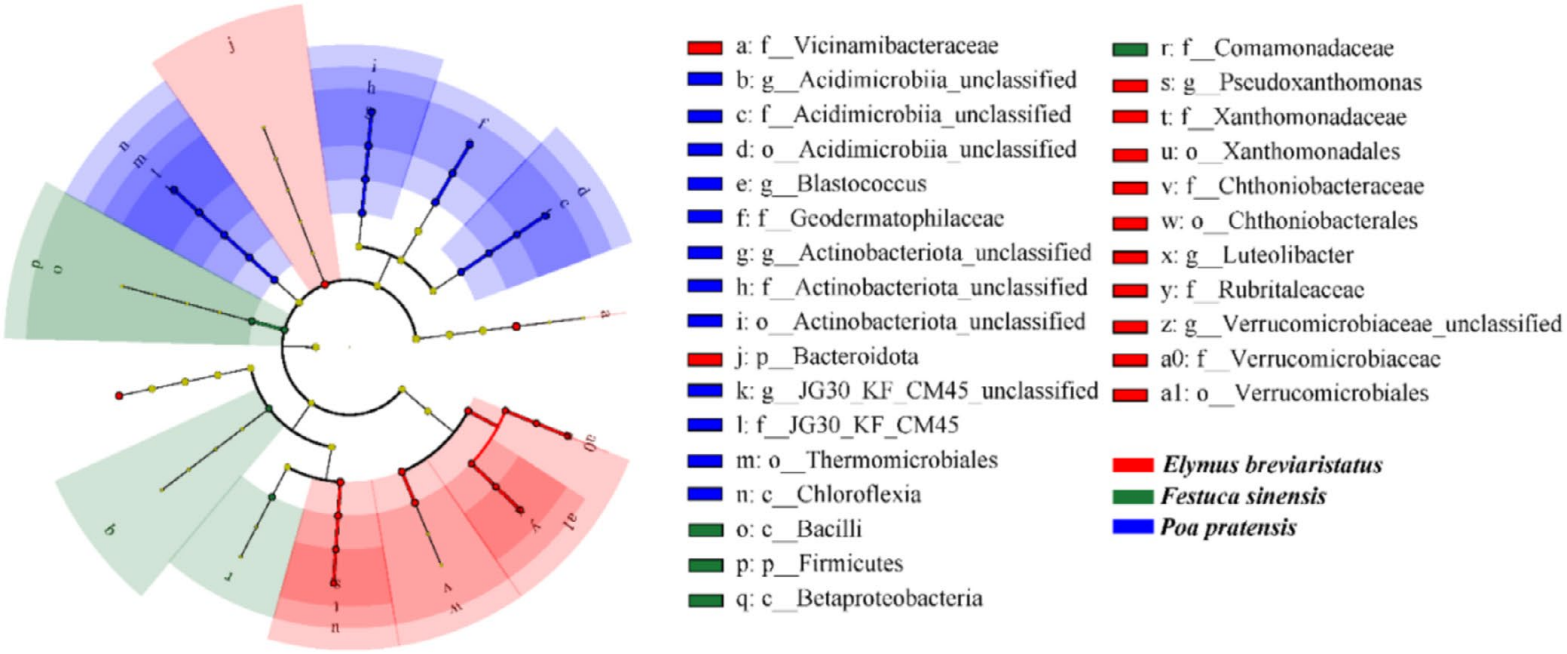
The cladogram shows significant differences between bacterial enrichment groups. Taxa with significant differences in abundance between different treatments are represented by colored dots, and cladogram circles represent phylogenetic taxa from phylum to genus. Only the LDA score 3 for bacterial was shown.

In all soil samples, the soil bacterial community was classified into 43 phyla. Among these, *Proteobacteria*, *Actinobacteria*, *Acidobacteria*, *Chloroflexi*, and *Gemmatimonadetes* emerged as the dominant phyla, constituting more than 80% of the total sequences. The top 10 phyla in relative abundance were evenly distributed among the three groups (Figure 3A). Among the four dominant bacteria with significant differences, *Chloroflexi* had a significantly higher relative abundance in the *Poa pratensis* monocropping grassland than in *Elymus breviaristatus* and *Festuca sinensis* monocropping (Figure 3B). *Verrucomicrobiota* had a significantly higher relative abundance in the *Elymus breviaristatus* monocropping than in *Festuca sinensis* and *Poa pratensis* monocropping (Figure 3C), while *Firmicutes* and *Fibrobacterota* had significantly higher relative abundances in the *Festuca sinensis* monocropping than in *Elymus breviaristatus* and *Poa pratensis* monocropping (Figure 3D,E). Although *Proteobacteria*, *Actinobacteria*, and *Acidobacteria* were not significantly different as dominant phyla (*p* > 0.05), their relative abundances exhibited regular patterns in rhizosphere soils of different plants. For example, the relative abundances of *Proteobacteria* and *Acidobacteria* were higher in *Elymus breviaristatus* than in *Festuca sinensis* and *Poa pratensis* , whereas the reverse was true for *Actinobacteria*, with *Poa pratensis* showing higher abundances than *Elymus breviaristatus* and *Festuca sinensis*. LEfSe analysis revealed distinct differences in bacterial communities among the three monocropping grasslands. Specifically, at the levels of phylum, class, order, family, and genus, certain bacterial taxa were significantly enriched in different monocropping grasslands (Figure 4). Most bacterial taxa were primarily concentrated in *Elymus breviaristatus* and *Poa pratensis* , with p__*Bacteroidota*, o__*Xanthomonadales*, and f__*Vicinamibacteraceae* playing important roles in E, and f__*Geodermatophilaceae*, g__*Blastococcus*, and s__*Blastococcus_unclassified* being the key microbial groups in *Poa pratensis* .

### 3.4 Bacterial Community Co‐Occurrence Network

**FIGURE 5:**
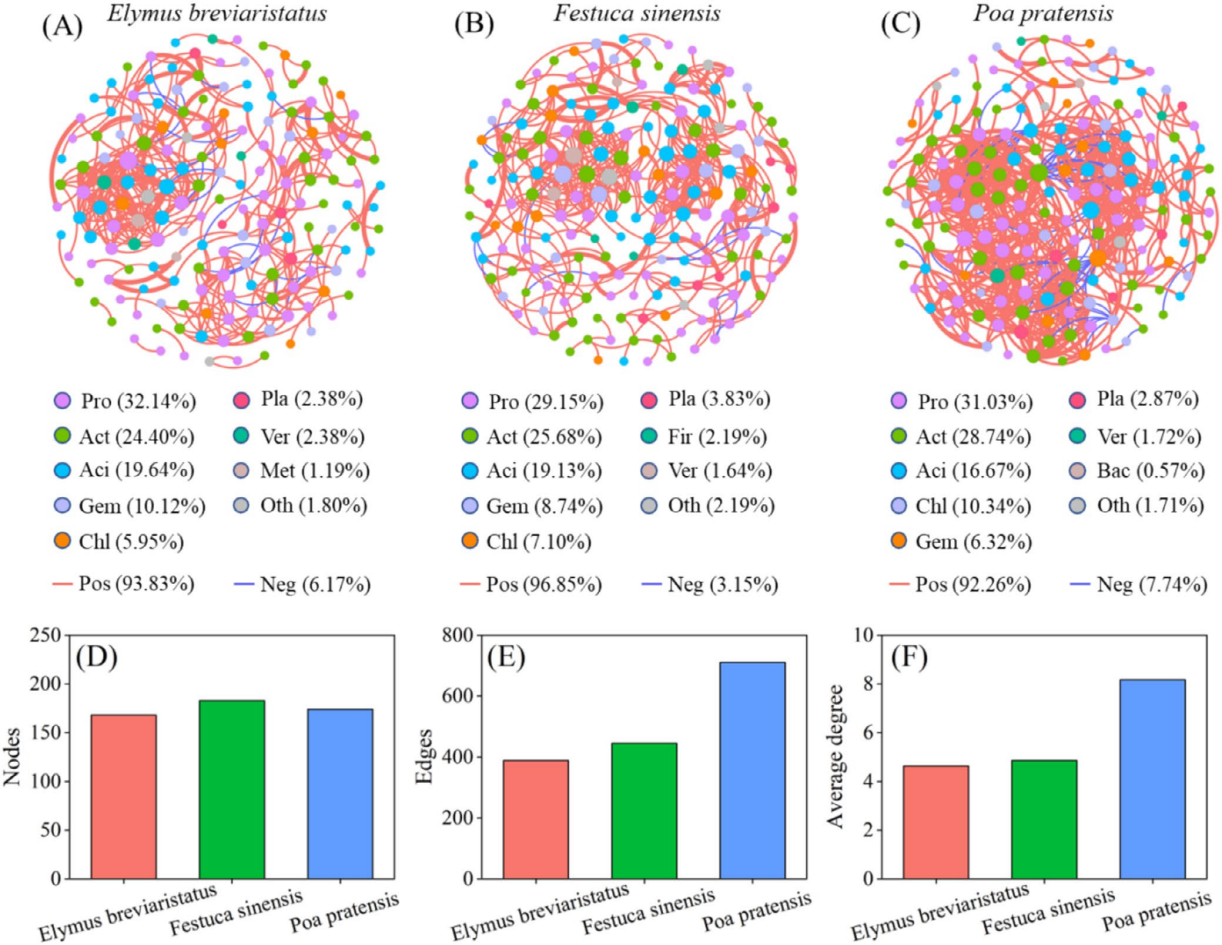
Co‐occurrence networks for bacterial species among different treatments. (A–C) represent the bacterial co‐occurrence networks based on pairwise Spearman's correlations between bacterial operational taxonomic units (OTUs) in *Elymus breviaristatus* , *Festuca sinensis* and *Poa pratensis* . (D–F) are the nodes, edges and average degree among different treatments.

To further evaluate the ecological patterns of rhizosphere soil microorganisms in different monocropping grasslands, we conducted a co‐occurrence network analysis. This network revealed the symbiotic patterns of soil bacterial communities (Figure 5). Overall, in the rhizosphere bacterial network, *Festuca sinensis* exhibited relatively high nodes (183), followed by *Poa pratensis* (174) and *Elymus breviaristatus* (168). *Poa pratensis* contained the most edges (711), with 92.26% being positive and 7.74% being negative. In contrast, *Elymus breviaristatus* had the fewest edges (389), with 93.83% being positive and 6.17% being negative. Using the number of connections and average degree to represent the complexity of the microbial network, *Poa pratensis* showed higher network complexity than *Elymus breviaristatus* and *Festuca sinensis*, indicating more intricate interspecific relationships and higher network stability. The average degree of *Poa pratensis* was higher than that of the other two treatments, suggesting stronger interconnections among the abundant OTUs in the *Poa pratensis* rhizosphere bacterial network. Additionally, the modularity values of bacteria among the three monocropping grasslands were all > 0.4, indicating that the constructed networks were modular.

### 3.5 Rhizosphere Soil Metabolites

**FIGURE 6:**
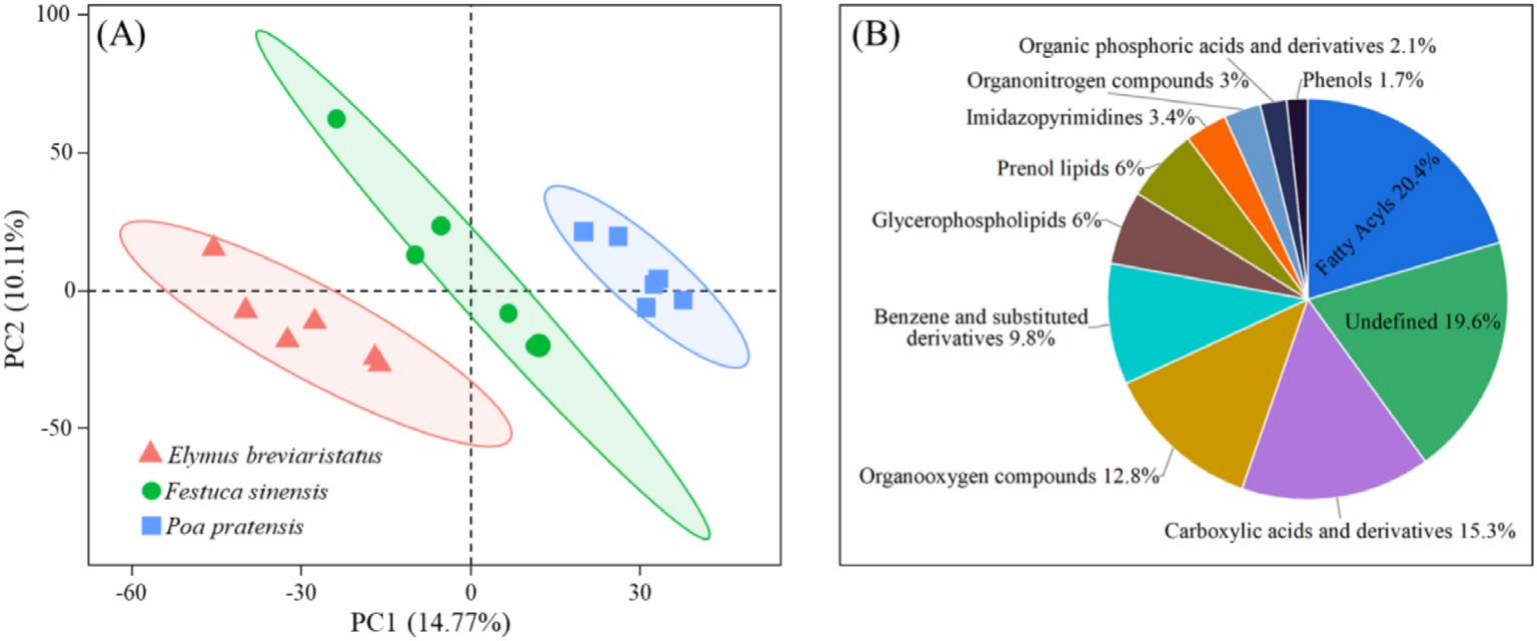
PLS‐DA score (A) and metabolite classification (B) of rhizosphere metabolites between different treatments.

In supervised models, principal component analysis (PCA) is used to evaluate the overall differences between various treatments. The results from PLS‐DA show that PC1 and PC2 account for 14.77% and 10.11% of the variance, respectively, and there is clear separation of the three sample groups along these axes (Figure 6A). This indicates significant differences in rhizosphere metabolites among the three different grasslands (*p* < 0.05). LC–MS analysis identified a total of 11,157 metabolites in the rhizosphere of perennial grasslands under both positive and negative ion conditions, with 286 annotated as secondary metabolites. Of these, 44 classes of secondary metabolites represent more than 1% of the total metabolite proportion, including Fatty Acyls (20.4%), Carboxylic acids and derivatives (15.3%), Organooxygen compounds (12.8%), Benzene and substituted derivatives (9.8%), Glycerophospholipids (6%), Prenol lipids (6%), Imidazopyrimidines (3.4%), Organonitrogen compounds (3%), Organic phosphoric acids and derivatives (2.1%), Phenols (1.7%), and Undefined compounds (19.6%) (Figure 6B).

**FIGURE 7:**
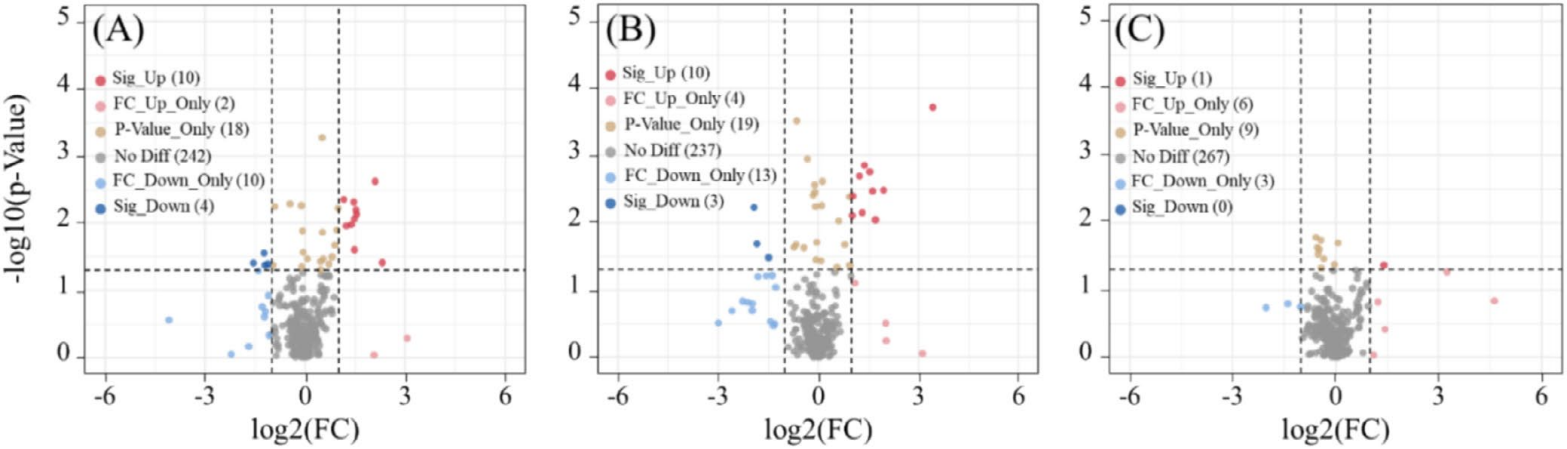
The volcanic plot of differential metabolites under different treatments. (A) Volcano plot of differential metabolites in group *Elymus breviaristatus* vs. *Festuca sinensis*. (B) Volcano plot of differential metabolites in group *Elymus breviaristatus* vs. *Poa pratensis* . (C) Volcano plot of differential metabolites in group *Festuca sinensis* vs. *Poa pratensis* .

To characterize these compounds metabolically, a VIP threshold of ≥ 1, *p* < 0.05, and FC > 2 or FC < 1/2 was applied for further analysis of annotated metabolites. In the *Elymus breviaristatus* vs. *Festuca sinensis* comparison, 14 metabolites with significant differences were identified. Among these, 10 metabolites were upregulated (primarily Organooxygen compounds and Carboxylic acids and derivatives), indicating higher concentrations in *Elymus breviaristatus* , while four metabolites were downregulated (mainly Fatty Acyls), suggesting reduced levels in *Elymus breviaristatus* (Figure 7A). In the *Elymus breviaristatus* vs. *Poa pratensis* comparison, 13 metabolites with significant differences were found. Here, 10 metabolites were upregulated (mainly Organooxygen compounds), showing higher concentrations in *Elymus breviaristatus* , while three metabolites were downregulated (mainly Fatty Acyls), indicating higher levels in *Poa pratensis* (Figure 7B). In the *Festuca sinensis* vs. *Poa pratensis* comparison, only one metabolite, 2‐Methylbenzaldehyde, was significantly upregulated (Figure 7C).

### 3.6 Enrichment Analysis of Metabolite KEGG

**FIGURE 8:**
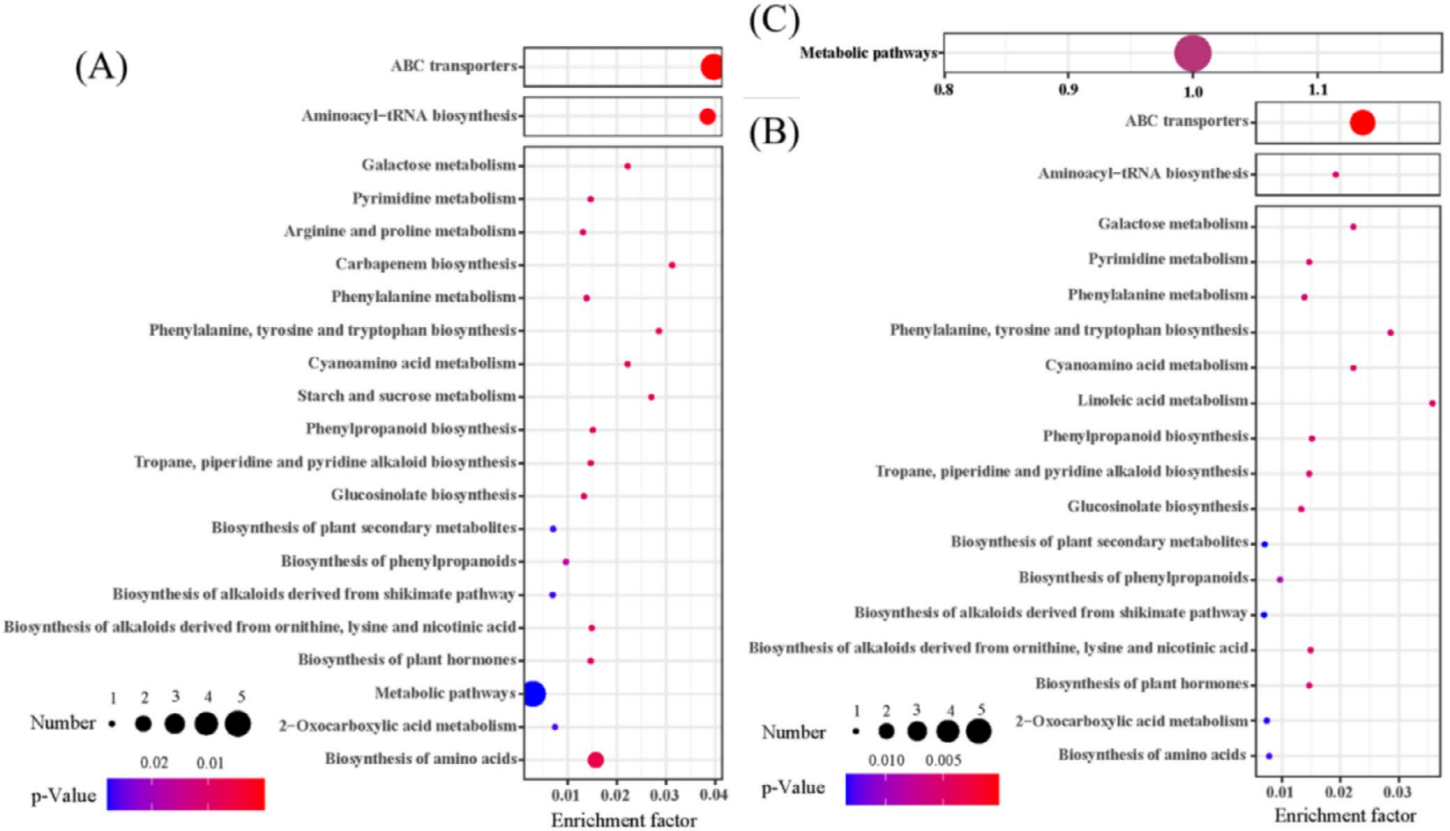
The KEGG pathway enrichment analysis of differential metabolites under different treatments. (A) KEGG enrichment scatter plot of differential metabolites in group *Elymus breviaristatus* vs. *Festuca sinensis*. (B) KEGG enrichment scatter plot of differential metabolites in group *Elymus breviaristatus* vs. *Poa pratensis* . (C) KEGG enrichment scatter plot of differential metabolites in group *Festuca sinensis* vs. *Poa pratensis* .

Further KEGG pathway enrichment analysis was conducted on the identified metabolites. The results show that the 14 differentially expressed metabolites in the *Elymus breviaristatus* versus *Festuca sinensis* comparison are enriched in 21 metabolic pathways (Figure 8A). The top three pathways based on P‐value in *Elymus breviaristatus* vs. *Festuca sinensis* are ABC transporters, Aminoacyl‐tRNA biosynthesis, and Biosynthesis of amino acids. For the 13 differentially expressed metabolites in the *Elymus breviaristatus* versus *Poa pratensis* comparison, KEGG enrichment analysis revealed a total of 18 metabolic pathways (Figure 8B). The top three pathways in *Elymus breviaristatus* versus *Poa pratensis* , based on P‐value, are ABC transporters, Linoleic acid metabolism, and Phenylalanine, tyrosine, and tryptophan biosynthesis. In the *Festuca sinensis* vs. *Poa pratensis* comparison, the differential metabolites are primarily enriched in the Metabolic pathways (Figure 8C). The relevant differentially expressed metabolites enriched in these pathways include Phenylalanine, Proline, Raffinose, Trehalose, Maltotriose, Uridine, and 2‐Methylbenzaldehyde, all of which show significant upregulation in each group (Table 2).

**TABLE 2:** Differential metabolites corresponding to major metabolic pathways of different treatments.

Group	Pathway	*p*	Metabolites
*Elymus breviaristatus* vs. *Festuca sinensis*	ABC transporters	< 0.001	Phenylalanine↑ Proline↑ Raffinose↑ Trehalose↑ Maltotriose↑
	Aminoacyl‐tRNA biosynthesis	< 0.001	Phenylalanine↑ Proline↑
	Biosynthesis of amino acids	< 0.01	Phenylalanine↑ Proline↑
	Pyrimidine metabolism	< 0.01	Uridine↑
*Elymus breviaristatus* vs. *Poa pratensis*	ABC transporters	< 0.001	Phenylalanine↑ Raffinose↑ Maltotriose↑
	Phenylalanine, tyrosine and tryptophan biosynthesis	< 0.001	Phenylalanine↑
	Pyrimidine metabolism	< 0.01	Uridine↑
*Festuca sinensis* vs. *Poa pratensis*	Metabolic pathways	< 0.05	2‐Methylbenzaldehyde↑

### 3.7 Drivers of Productivity Differences Between Monocropping Grasslands

**FIGURE 9:**
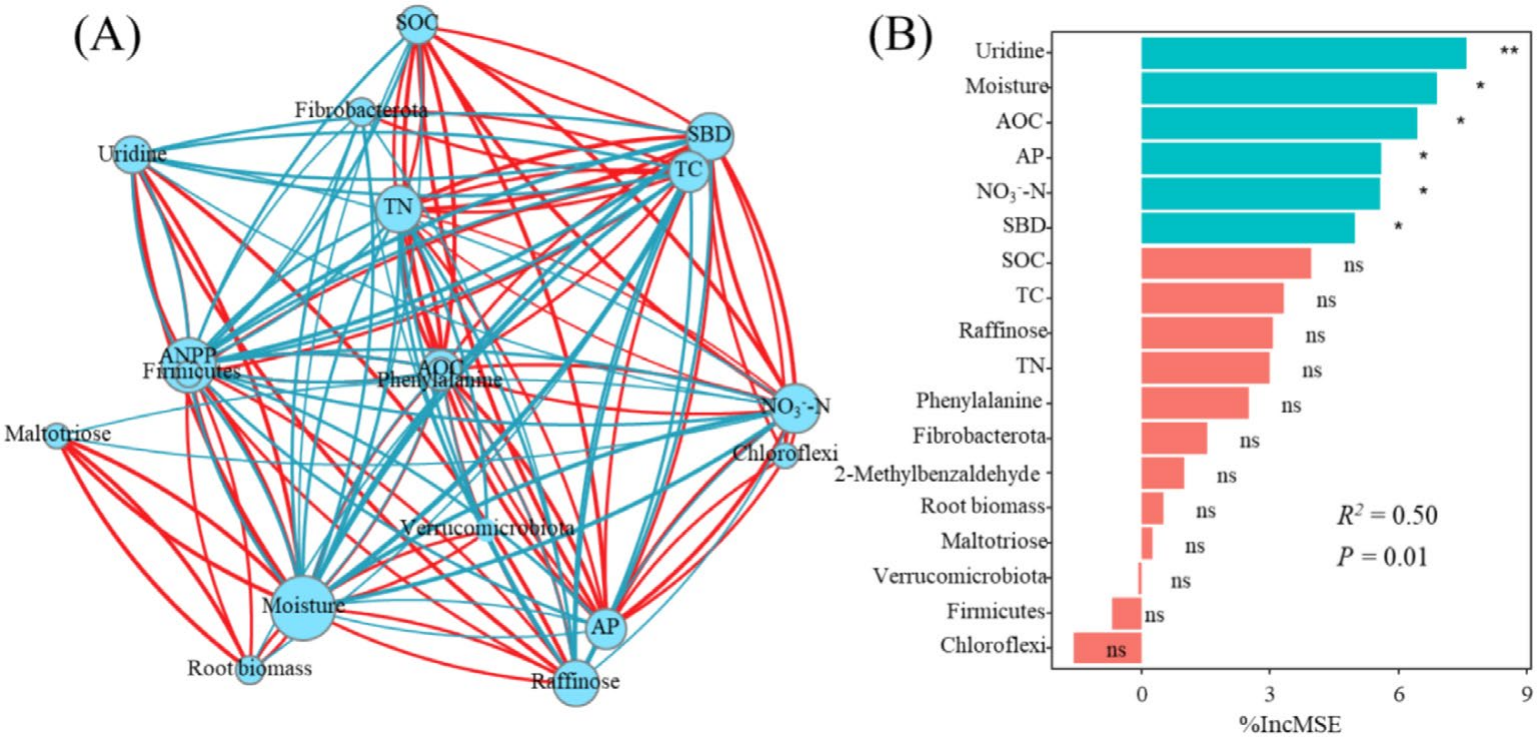
Co‐occurrence analysis between soil properties, soil bacteria, and root metabolites (A). Random forest analysis identified the key physicochemical factors that dominate changes in ANPP (B). The node size is related to the number of connections, the red or blue lines represent positive or negative co‐occurrence. ANPP, above‐ground net primary productivity; AOC, soil active organic carbon; AP, soil available phosphorus; NO_3_^−^‐N, soil nitrate‐nitrogen; SBD, soil bulk density; SOC, soil organic carbon; TC, soil total carbon; TN, soil total nitrogen.

Pearson correlation analysis was conducted between differential metabolites, bacterial communities, soil physicochemical properties, and productivity. The results indicate that the productivity of monocropping grasslands is significantly correlated with soil physicochemical properties (Moisture, SBD, TN, NO_3_^−^‐N, AOC, AP, TC, SOC), root biomass, differential metabolites (Uridine, Raffinose and Phenylalanine) and microbial (*Fibrobacterota*) contents (Figure 9A). Random forest analysis further identified Uridine, Moisture, AOC, AP, NO_3_^−^‐N, and SBD as key factors driving productivity changes in perennial monocropping grasslands (*R* ^2^ = 0.50, *p* = 0.01) (Figure 9B).

## 4 Discussion

### 4.1 Changes of Productivity of Different Monocropping Grasslands

Plant productivity, a crucial ecological characteristic closely linked to nutrient cycling, energy flow, and carbon cycling, is significantly indicated by aboveground biomass as a key measure of community productivity (An et al. 2024; Xu et al. 2019). In this study, the productivity of different monocropping grasslands was significantly different, with the productivity from high to low being *Elymus breviaristatus* (589.17 g·m^−2^) > *Festuca sinensis* (480.43 g·m^−2^) > *Poa pratensis* (420.67 g·m^−2^), which may be related to the biological characteristics and phenological period of the three plants. Among them, *Elymus breviaristatus* was an overgrowing grass with tall plants, while *Festuca sinensis* and *Poa pratensis* were small and weak, so the aboveground biomass of *Elymus breviaristatus* was significantly higher than that of *Festuca sinensis* and *Poa pratensis* . Research has shown that phenology can have species‐specific effects on the aboveground net primary productivity of grasslands. Changes in plant phenological phases can shorten or extend the length of the growing season, thereby altering the duration of photosynthesis and ultimately affecting plant productivity (Yang and Zhao 2023). This indicates that interspecific phenological differences influence the productivity of different grassland species (An et al. 2024).

### 4.2 Changes of Soil Physicochemical Properties of Different Monocropping Grasslands

Soil, as one of the key drivers of vegetation succession, serves as the site for plant growth and development; it plays a crucial role in nutrient cycling within grassland ecosystems. The physicochemical properties of soil—such as moisture content, bulk density, pH, soil organic matter, total nutrients, and total carbon—are major factors affecting forage yield and quality (Li et al. 2018). Different plant species have varying nutrient requirements and can tolerate different nutrient levels in the soil. This means that a rich nutrient supply can support a greater diversity of plant species (Olff and Ritchie 1998). In this study, significant differences were observed in TC, TN, SOC, AOC, NO_3_^−^‐N, AP, Moisture, and SBD, while soil pH and TP content did not show significant differences. This indicates that different forage varieties have varying degrees of soil adaptability. The results of this study showed that soil nutrient content was negatively correlated with productivity. Inconsistent with previous research, this difference may be due to differences in vegetation types, soil heterogeneity, and climatic conditions. These factors interact with soil nutrients in complex ways, leading to variations in the relationship between plant productivity and soil nutrients (Jing et al. 2019). At certain levels, increasing soil nutrient levels may start to negatively affect plant growth and diversity.

### 4.3 Changes of Bacterial Diversity and Community Structure in Different Monocropping Grassland

Microorganisms are crucial for plant growth and health, aiding in nutrient absorption, supporting tolerance to abiotic stress, and protecting hosts from plant pathogens (Hartman et al. 2018). Factors influencing soil microbial diversity include natural elements such as soil type, climate, and vegetation, as well as various anthropogenic factors (Tohtahun et al. 2024). Studies have shown that vegetation type is a key determinant of microbial community structure. Different plant litter and root exudates can impact the types, quantities, and distribution of microbial communities, as well as affect the soil microenvironment, shaping the soil microbial community (Han et al. 2018). Additionally, soil microbial communities can directly or indirectly drive organic matter decomposition and transformation, influencing plant growth and development, and thereby affecting the composition and structure of plant communities (Schimel et al. 1999). In this study, except that Chao1 and Observed OTUs indices at the class level were significantly different in different monoculture grasslands, the other α diversity was not significantly different, with *Festuca sinensis* grasslands being significantly higher than *Elymus breviaristatus* and *Poa pratensis* .

Soil cultivation and plant species are major factors influencing soil microbial community structure (Yan et al. 2022). Soils under different vegetation restoration types show some similarity in bacterial phyla composition at the phylum level, with differences mainly in relative abundance. In this study, the dominant bacterial phyla in different monocropping grasslands were *Proteobacteria* (25.88% ~ 16.50%), *Actinobacteria* (22.97% ~ 26.38%), *Acidobacteria* (15.27% ~ 17.18%), *Chloroflexi* (7.57% ~ 8.92%), and *Gemmatimonadetes* (7.81% ~ 8.03%), consistent with previous findings. However, different forage types did not significantly alter the relative abundance of dominant phyla, except for *Chloroflexi*. This is because, compared to weeds and legumes, grasses have a lesser impact on microbial community structure (Ladygina and Hedlund 2010). At the community level, a higher abundance of dominant bacterial phyla related to soil denitrification processes (such as *Proteobacteria* and *Firmicutes*) suggests a potentially stronger denitrification process in the soil (Wei et al. 2015). In this study, the relative abundance of *Firmicutes* was significantly higher in *Festuca sinensis* compared to *Elymus breviaristatus* and *Poa pratensis* . Microbial taxa can be classified according to classical life history strategies into K‐strategists (oligotrophic organisms) and R‐strategists (copiotrophic organisms). K‐strategists dominate in undisturbed and mature ecosystems, effectively utilizing stable substrates at the cost of slower growth rates. In contrast, R‐strategists have higher growth rates and depend on readily available substrates (Morrissey et al. 2022). At the phylum level, *Acidobacteria*, *Actinobacteria*, *Verrucomicrobia*, and *Chloroflexi* are classified as potential K‐strategists (oligotrophic bacteria), while *Firmicutes*, *Gemmatimonadetes*, and *Bacteroidetes* are classified as R‐strategists (copiotrophic bacteria). *Chloroflexi*, being K‐strategists, can fix and absorb sulfate, which can significantly inhibit plant growth (Hu et al. 2022). Studies by Lu et al. (2018) have shown that reductions in *Actinobacteria* and *Chloroflexi* are crucial for improving soil quality and plant yield. *Verrucomicrobiota* are important contributors to nitrogen fixation, polymer‐carbon substrate degradation, and polyphenol degradation. In this study, the relative abundance of *Actinobacteria* and *Chloroflexi* was lowest in *Elymus breviaristatus* , while *Verrucomicrobiota* abundance was highest in *Elymus breviaristatus* , correlating with the highest aboveground biomass in *Elymus breviaristatus* .

Co‐occurrence network analysis can visualize the complex relationships within soil microbial communities, revealing the potential ecological roles of different soil microbial taxa (Tian et al. 2022). The number of nodes and connections in a molecular ecological network indicates the scale and complexity of relationships. Positive correlations represent symbiotic or mutualistic relationships, while negative correlations denote competitive or antagonistic interactions (Yan et al. 2022). Results from this study show that *Poa pratensis* had higher connectivity and average degree, indicating that bacterial taxa in its rhizosphere appeared more frequently, and the relationships among OTUs in the rhizosphere bacterial network were stronger, leading to greater complexity and stability. Additionally, the network connections among bacteria in *Festuca sinensis* (96.85%) were more positively correlated compared to *Elymus breviaristatus* (93.83%) and *Poa pratensis* (92.26%), suggesting stronger cooperative relationships in the *Festuca sinensis* bacterial community. Such positive cooperation may enhance community resilience in changing environments, as microbial interaction networks can buffer environmental disturbances (Zhao, Ma, et al. 2022). Furthermore, the number of nodes and modularity in the *Festuca sinensis* (183, 0.81) rhizosphere bacterial network were higher than in *Elymus breviaristatus* (168, 0.78) and *Poa pratensis* (174, 0.67). Module hubs and connectors are considered key species, playing crucial roles in the network structure (Tian et al. 2022). These findings indicate that *Festuca sinensis* soil exhibits stronger niche differentiation and greater sensitivity to environmental changes, consistent with previous research (Zhang et al. 2018).

### 4.4 Changes of Soil Metabolites in Different Monocropping Grassland

Rhizosphere respiration and metabolites are key drivers influencing soil physicochemical and biological properties (Philippot et al. 2013). The composition and quantity of metabolites directly affect the dominant microbial taxa and the composition of bacterial and fungal communities. These underground dynamics can be beneficial or detrimental to plant health and development, leading to changes in plant community structure and productivity (Zhou et al. 2019). Metabolic pathways such as ABC transporters, Aminoacyl‐tRNA biosynthesis, and pyrimidine metabolism play a crucial role in plants, affecting plant growth and development, and adaptability to the environment (Peng et al. 2023). Pyrimidine metabolism also plays a critical role in soil, mainly involving the metabolic activities of soil microorganisms and their impact on plant growth (Yuan et al. 2024). It affects plant growth and health directly and, through microbial activity, converts organic carbon to inorganic carbon, providing essential nutrients and indirectly affecting plants (Su et al. 2023). In this study, the metabolites enriched in these pathways include Phenylalanine, Proline, Raffinose, Maltotriose, and Uridine. These metabolites play a key role in plant response to environmental stress through their osmoregulatory effects and properties as antioxidants, helping plants adapt to and resist various adverse environmental conditions, thereby protecting plants from injury and promoting plant growth and development (Dong, Sun, et al. 2020; Feng et al. 2023). These differential metabolites were significantly upregulated in both *Elymus breviaristatus* versus *Festuca sinensis* and *Elymus breviaristatus* versus *Poa pratensis* , indicating their higher abundance in *Elymus breviaristatus* . This difference may be attributed to the root characteristics (morphological and physiological) of the forage species, as well as variations in soil physicochemical properties, which may lead to significant differences in plant growth patterns and could be a reason for the metabolic differences among the three forage species (Siri‐Prieto et al. 2020). Uridine, phenylalanine, and raffinose showed a significant positive correlation with ANPP, and notably, Phenylalanine, Maltotriose, Uridine, and Raffinose exhibited consistent abundance changes under the same conditions, suggesting a potential relationship with ANPP.

### 4.5 The Interaction Between Soil Nutrients, Rhizosphere Metabolites, and Microorganisms Determines the Productivity of Monocropping Grassland

Plant species can influence the quantity and quality of organic matter in the soil, which in turn has a significant impact on soil microbial growth, activity, and the accumulation of microbial products in the soil (Jia et al. 2022). Soil microorganisms can affect plant primary productivity either directly (through symbionts and pathogens) or indirectly (Li et al. 2023). *Proteobacteria* and *Bacteroidetes* can enhance plant and plant community productivity by increasing the availability of nitrogen and phosphorus or by suppressing soil pathogens (Zhou et al. 2019). Environmental factors such as soil pH, moisture, organic carbon, and total nitrogen significantly affect the abundance, diversity, structure, and composition of soil bacterial communities (Wang et al. 2024). *Gemmatimonadota* and *Actinobacteria* have been identified as key factors in predicting critical soil properties, particularly SOC, DOC, AN, and TN (Yuan et al. 2024). Consistent with previous studies, this research found that soil moisture significantly impacts bacterial diversity, while TC, AOC, TN, NO_3_^−^‐N, and SBD significantly influence bacterial community composition. Rhizosphere metabolites provide readily available carbon and nutrients for the growth and reproduction of rhizosphere microorganisms (Ding et al. 2021).

Additionally, rhizosphere metabolites not only directly regulate the microbiome but also alter the rhizosphere environment, leading to indirect effects. For example, organic acids in metabolites can change the pH of rhizosphere soil, thereby influencing the diversity, activity, and function of the rhizosphere microbiome (Ding et al. 2021; Morriën et al. 2017). Bi et al. (2022) discovered that rhizosphere metabolites released by *P. sylvestris* can alter the diversity of rhizosphere microbial communities. Zhang et al. (2022) indicated that with increasing planting years, the accumulation of saponins in oil tea flower soil affects the diversity and function of soil microorganisms. *Artemisia annua* secretes carbohydrates, amino acids, and organic acids, which can increase the C and N content in the rhizosphere, leading to the accumulation of microbes that accelerate plant growth, such as Sphingomonas, Sphingobium, and Saprotrophic fungi (Shi et al. 2021). Higher NO_3_‐ availability can weaken the colonization of *Fusarium* and reduce the damage *Fusarium* acid inflicts on plant tissues (Tong, Zheng, et al. 2024). Plants regulate the composition and concentration of metabolites through genetic mechanisms, which then influence microbial abundance. Microbes, in turn, provide feedback to plants by promoting growth and development (Muhammad Aslam et al. 2022). Some rhizosphere metabolites can directly encourage or inhibit pathogens. The compounds released by plant roots are chemically diverse, primarily including sugars, organic acids, and other secondary metabolites, which can regulate soil biochemical processes and ultimately stimulate changes in rhizosphere soil metabolites (Haichar et al. 2014). The dynamic changes in the composition and relative abundance of soil metabolites profoundly affect the physicochemical properties of the rhizosphere soil (Wang et al. 2023). In this study, metabolites significantly impacted soil physicochemical properties (SBD, Moisture, TC, TN, NO_3_^−^‐N, AP, AOC, SOC). Gramineous plants are rich in carbohydrates, which are secreted into the soil through the roots, contributing to the accumulation of SOM (Ling et al. 2022). A larger ecological niche breadth indicates a more extensive resource metabolism, which enhances resource use efficiency and holds greater potential to promote plant growth and health in low‐fertility soils (Jiang et al. 2023). Rhizosphere metabolites, which are unstable, influence rhizosphere microorganisms' nutrient limitations by conveying chemical signals or providing substrates (Che et al. 2024). Changes in metabolites corresponding to soil nutrient availability regulate the selective pressures on microbial community structure and stimulate microbial activity. Generally, plants secrete organic compounds (such as organic acids, sugars, flavonoids, and amino acids) to recruit beneficial microorganisms, thus exerting strong selective pressures on microbial assembly (Wang et al. 2022). Yu et al. (2021) have indicated that under nitrogen‐limited soil conditions, maize root exudates are modified to attract beneficial soil microorganisms, thereby enhancing plant nitrogen availability. Sasse et al. (2017) also observed that the composition of root exudates significantly affects microbial community metabolism and function. Our study found that metabolites had minimal impact on the rhizosphere microbial community, which contrasts with previous research. This discrepancy might be due to the fact that the correlation between microorganisms and metabolites may not be a direct effect of metabolites on microorganisms, but rather a result of interactions among microorganisms themselves. Additionally, rhizosphere microorganisms can activate necessary nutrients by secreting extracellular enzymes (Jing et al. 2019). Ecosystems function as integrated wholes, where individuals evolve within the context of the whole system. In the long‐term co‐evolution between plants and microorganisms, both parties balance their inputs and outputs. Opportunistic cooperative strategies are retained when they benefit both sides and tend to exist more in a networked form rather than direct interactions (Hallam and McCutcheon 2015). Moreover, metabolites not only provide ample nutrients to microorganisms but also alter the physicochemical properties of the rhizosphere, significantly impacting nutrient conditions in the rhizosphere. Soil microbial communities and soil nutrient status are directly related to the metabolites secreted by plants (Wang et al. 2023). Secondary metabolites can influence bacterial growth and, consequently, overall metabolism. Our results align with previous findings showing a significant negative correlation between *Firmicutes* and some key metabolites like L‐valine. Additionally, significant negative correlations were observed between *Gemmatimonadota*, *Nitrospirota*, and *Myxococcota* with depleted metabolites (Yuan et al. 2024). Amino acids, as part of the rhizosphere carbon‐nitrogen pool, are considered essential microbial substrates, unlike sugars and organic acids. However, high levels of certain amino acids, such as L‐valine, may be detrimental to some bacteria (Liu et al. 2021). We found that Phenylalanine and Uridine decreased the relative abundance of *Firmicutes*.

The results of this study may vary due to factors such as soil type, past soil management, and the season or timing of the study. Further validation of these findings could be achieved through experiments conducted across different seasons, locations, and key developmental stages of various grass species throughout their growth periods. Additionally, comparing differences in grass genotypes and soil enzymes could provide deeper insights into the relationships between soil nutrients, rhizosphere microorganisms, and metabolites, and reveal the dominant factors contributing to productivity differences among different monocropping grasslands. Finally, 16S amplicon sequencing has many limitations. For example, 16S universal primers may lead to an underestimation of the number of specific populations; the 16S copy number of different strains is different, and the 16S sequence within strains is also different, which makes absolute quantification difficult; OTUs analysis may result in some sequences not being accurately annotated; 16S rRNA sequencing usually only provides taxonomic information down to the genus or species level, not more detailed species‐level information; data disclosure and sharing can help to standardize microbiota research and data analysis and increase data comparability, but there are still challenges in this process. It can be further studied by metagenomic or macro‐transcriptomics.

## 5 Conclusions

This study provides, for the first time, an analysis of the impact of three commonly used *Poaceae* grasses in cultivated grasslands on rhizosphere metabolites on the Qinghai –Tibet Plateau. It offers a comprehensive assessment of the effects of rhizosphere soil physicochemical properties, soil bacterial communities, and metabolites on the productivity of perennial cultivated grasslands. The results indicate significant differences in productivity, soil nutrients, bacterial community structure, and relative abundance of certain phyla among the three monocropping grasslands on the Qinghai –Tibet Plateau. Untargeted metabolomics reveals significant metabolic differences between grasslands *Elymus breviaristatus* and *Festuca sinensis*, *Poa pratensis* , while similarities exist in metabolic patterns between grasslands *Festuca sinensis* and *Poa pratensis* . Identified rhizosphere metabolites, particularly Uridine, serve as metabolic markers. Key soil physicochemical variables affecting the productivity of monocropping grasslands include SBD, AP, Moisture, AOC, and NO_3_^−^‐N, whereas the principal rhizosphere metabolite influencing productivity is Uridine. Overall, our findings underscore the close correlations between the productivity of cultivated grasslands and soil bacterial communities, soil physicochemical properties, and metabolites.

## Author Contributions

**Xiaofang Zhang:** conceptualization (equal), data curation (equal), investigation (equal), software (equal), writing – original draft (equal), writing – review and editing (equal). **Yuzhen Liu:** visualization (equal), writing – review and editing (equal). **Quan Cao:** data curation (equal), formal analysis (equal), investigation (equal). **Zengzeng Yang:** data curation (equal), formal analysis (equal), investigation (equal). **Zehang Yu:** data curation (equal), formal analysis (equal). **Caidi Li:** data curation (equal), formal analysis (equal), investigation (equal). **Chunping Zhang:** data curation (equal), investigation (equal), methodology (equal), writing – review and editing (equal). **Quanmin Dong:** conceptualization (equal), funding acquisition (equal), methodology (equal), project administration (equal), writing – review and editing (equal).

## Conflicts of Interest

The authors declare no conflicts of interest.

## Data Availability

Data is available at http://datadryad.org/stash/share/ijp2o5DIP1ka8hjBlV8GNJf53CyfiMkxcUPcvgFxUok.

## References

[ece371149-bib-0001] An, S. , X. Q. Chen , F. J. Li , et al. 2024. “Long‐Term Species‐Level Observations Indicate the Critical Role of Soil Moisture in Regulating China's Grassland Productivity Relative to Phenological and Climatic Factors.” Science of the Total Environment 929: 172553. 10.1016/j.scitotenv.2024.172553. 38663615

[ece371149-bib-0002] Arvidsson, J. 1999. “Nutrient Uptake and Growth of Barley as Affected by Soil Compaction.” Plant and Soil 208, no. 1: 9–19.

[ece371149-bib-0003] Bakker, M. , D. Manter , A. Sheflin , T. Weir , and J. Vivanco . 2012. “Harnessing the Rhizosphere Microbiome Through Plant Breeding and Agricultural Management.” Plant and Soil 360: 1–13. 10.1007/s11104-012-1361-x.

[ece371149-bib-0004] Bi, B. Y. , Y. Yuan , H. Zhang , Z. H. Wu , Y. Wang , and F. P. Han . 2022. “Rhizosphere Soil Metabolites Mediated Microbial Community Changes of *Pinus sylvestris* var. Mongolica Across Stand Ages in the Mu us Desert.” Applied Soil Ecology 169: 104222. 10.1016/j.apsoil.2021.104222.

[ece371149-bib-0005] Chaudhary, D. R. , R. Gautam , B. Yousuf , A. Mishra , and B. Jha . 2015. “Nutrients, Microbial Community Structure and Functional Gene Abundance of Rhizosphere and Bulk Soils of Halophytes.” Applied Soil Ecology 91: 16–26. 10.1016/j.apsoil.2015.02.003.

[ece371149-bib-0006] Che, J. L. , Y. Q. Wu , H. Yang , et al. 2024. “Metabolites of Blueberry Roots at Different Developmental Stages Strongly Shape Microbial Community Structure and Intra‐Kingdom Interactions at the Root‐Soil Interface.” Science of the Total Environment 947: 174333. 10.1016/j.scitotenv.2024.174333. 38945231

[ece371149-bib-0007] Ding, X. W. , K. H. Liu , Q. Y. Yan , et al. 2021. “Sugar and Organic Acid Availability Modulate Soil Diazotroph Community Assembly and Species Co‐Occurrence Patterns on the Tibetan Plateau.” Applied Microbiology and Biotechnology 105: 8545–8560. 10.1007/s00253-021-11629-9. 34661705

[ece371149-bib-0008] Dong, N. Q. , Y. W. Sun , T. Guo , et al. 2020. “UDP‐Glucosyltransferase Regulates Grain Size and Abiotic Stress Tolerance Associated With Metabolic Flux Redirection in Rice.” Nature Communications 11, no. 1: 2629. 10.1038/s41467-020-16403-5. PMC7250897 32457405

[ece371149-bib-0009] Dong, R. Z. , X. L. Wang , Y. L. Wang , et al. 2023. “Differences in Soil Microbial Communities With Successional Stage Depend on Vegetation Coverage and Soil Substrates in Alpine Desert Shrublands.” Plant and Soil 485: 1–20. 10.21203/rs.3.rs-1803584/v1.

[ece371149-bib-0010] Dong, S. K. , Z. H. Shang , J. X. Gao , and R. B. Boone . 2020. “Enhancing Sustainability of Grassland Ecosystems Through Ecological Restoration and Grazing Management in an Era of Climate Change on Qinghai‐Tibetan Plateau.” Agriculture, Ecosystems & Environment 287: 106684. 10.1016/j.agee.2019.106684.

[ece371149-bib-0011] Feng, Z. W. , X. L. Xie , P. D. Wu , et al. 2023. “Phenylalanine‐Mediated Changes in the Soil Bacterial Community Promote Nitrogen Cycling and Plant Growth.” Microbiological Research 275: 127447. 10.1016/j.micres.2023.127447. 37441843

[ece371149-bib-0012] Haichar, F. e. Z. , C. Santaella , T. Heulin , and W. Achouak . 2014. “Root Exudates Mediated Interactions Belowground.” Soil Biology and Biochemistry 77: 69–80. 10.1016/j.soilbio.2014.06.017.

[ece371149-bib-0013] Hallam, S. , and J. McCutcheon . 2015. “Microbes Don't Play Solitaire: How Cooperation Trumps Isolation in the Microbial World.” Environmental Microbiology Reports 7: 26–28. 10.1111/1758-2229.12248. 25721597

[ece371149-bib-0014] Han, D. X. , N. Wang , X. Sun , Y. B. Hu , and F. J. Feng . 2018. “Biogeographical Distribution of Bacterial Communities in Changbai Mountain, Northeast China.” MicrobiologyOpen 7, no. 2: 1–9. 10.1002/mbo3.529. PMC5911996 29446229

[ece371149-bib-0015] Hartman, K. , M. van der Heijden , R. Wittwer , S. Banerjee , J. C. Walser , and K. Schlaeppi . 2018. “Cropping Practices Manipulate Abundance Patterns of Root and Soil Microbiome Members Paving the Way to Smart Farming.” Microbiome 6, no. 1: 1–14. 10.1186/s40168-017-0389-9. 29338764 PMC5771023

[ece371149-bib-0016] Hu, P. L. , W. Zhang , Y. Kuzyakov , et al. 2022. “Linking Bacterial Life Strategies With Soil Organic Matter Accrual by Karst Vegetation Restoration.” Soil Biology and Biochemistry 177, no. 4: 108925. 10.1016/j.soilbio.2022.108925.

[ece371149-bib-0017] Huang, X. M. , S. R. Liu , H. Wang , Z. D. Hu , Z. G. Li , and M. Ye . 2014. “Changes of Soil Microbial Biomass Carbon and Community Composition Through Mixing Nitrogen‐Fixing Species With Eucalyptus Urophylla in Subtropical China.” Soil Biology and Biochemistry 73: 42–48. 10.1016/j.soilbio.2014.01.021.

[ece371149-bib-0018] Jia, B. , L. Jia , Y. M. Zhang , X. M. Mou , and X. G. Li . 2022. “Leguminous *Caragana korshinskii* Evidently Enhances Microbial Necromass Carbon Accumulation in Dryland Soils.” Catena 215: 106342. 10.1016/j.catena.2022.106342.

[ece371149-bib-0019] Jiang, M. T. , M. Delgado‐Baquerizo , M. M. Yuan , et al. 2023. “Home‐Based Microbial Solution to Boost Crop Growth in Low‐Fertility Soil.” New Phytologist 239, no. 2: 752–765. 10.1111/nph.18943. 37149890

[ece371149-bib-0020] Jiang, Z. , J. M. Wang , K. Q. Cao , et al. 2024. “Foliar Application of Selenium and Gibberellins Reduce Cadmium Accumulation in Soybean by Regulating Interplay Among Rhizosphere Soil Metabolites, Bacteria Community and Cadmium Speciation.” Journal of Hazardous Materials 476: 134868. 10.1016/j.jhazmat.2024.134868. 38897119

[ece371149-bib-0021] Jing, X. , X. Chen , J. Y. Fang , et al. 2019. “Soil Microbial Carbon and Nutrient Constraints Are Driven More by Climate and Soil Physicochemical Properties Than by Nutrient Addition in Forest Ecosystems.” Soil Biology and Biochemistry 141: 107657. 10.1016/j.soilbio.2019.107657.

[ece371149-bib-0022] Koprivova, A. , and S. Kopriva . 2022. “Plant Secondary Metabolites Altering Root Microbiome Composition and Function.” Current Opinion in Plant Biology 67: 102227. 10.1016/j.pbi.2022.102227. 35525222

[ece371149-bib-0023] Ladygina, N. , and K. Hedlund . 2010. “Plant Species Influence Microbial Diversity and Carbon Allocation in the Rhizosphere.” Soil Biology and Biochemistry 42: 162–168. 10.1016/j.soilbio.2009.10.009.

[ece371149-bib-0024] Li, D. , C. R. Zhou , Y. L. Wu , et al. 2022. “Nanoselenium Integrates Soil‐Pepper Plant Homeostasis by Recruiting Rhizosphere‐Beneficial Microbiomes and Allocating Signaling Molecule Levels Under cd Stress.” Journal of Hazardous Materials 432: 128763. 10.1016/j.jhazmat.2022.128763. 35349848

[ece371149-bib-0025] Li, N. , N. Zhao , S. X. Xu , et al. 2023. “Accumulation of Microbial Necromass Carbon and Its Contribution to Soil Organic Carbon in Artificial Grasslands of Various Vegetation Types.” European Journal of Soil Biology 119: 103573. 10.1016/j.ejsobi.2023.103573.

[ece371149-bib-0026] Li, W. , J. L. Wang , X. J. Zhang , S. L. Shi , and W. X. Cao . 2018. “Effect of Degradation and Rebuilding of Artificial Grasslands on Soil Respiration and Carbon and Nitrogen Pools on an Alpine Meadow of the Qinghai‐Tibetan Plateau.” Ecological Engineering 111: 134–142. 10.1016/j.ecoleng.2017.10.013.

[ece371149-bib-0027] Li, Y. Y. , S. K. Dong , L. Wen , X. X. Wang , and Y. Wu . 2014. “Soil Carbon and Nitrogen Pools and Their Relationship to Plant and Soil Dynamics of Degraded and Artificially Restored Grasslands of the Qinghai–Tibetan Plateau.” Geoderma 213: 178–184. 10.1016/j.geoderma.2013.08.022.

[ece371149-bib-0028] Ling, N. , T. T. Wang , and Y. Kuzyakov . 2022. “Rhizosphere Bacteriome Structure and Functions.” Nature Communications 13, no. 1: 836. 10.21203/rs.3.rs-274143/v1. PMC8837802 35149704

[ece371149-bib-0029] Liu, Q. , Z. Q. Pang , H. R. Sun , et al. 2024. “Unveiling the Maize‐Benefit: Synergistic Impacts of Organic‐Inorganic Fertilizer Cooperation on Rhizosphere Microorganisms and Metabolites.” Applied Soil Ecology 193: 105171. 10.1016/j.apsoil.2023.105171.

[ece371149-bib-0030] Liu, Y. , J. T. Han , A. Wilson , L. O'Sullivan , and C. Haney . 2021. “Amino Acid Availability Determines Plant Immune Homeostasis in the Rhizosphere Microbiome.” MBio 14, no. 2: e03424‐22. 10.1128/mbio.03424-22. PMC10127609 36786577

[ece371149-bib-0031] Liu, Z. Q. , S. J. Li , N. Liu , G. Q. Huang , and Q. Zhou . 2022. “Soil Microbial Community Driven by Soil Moisture and Nitrogen in Milk Vetch (*Astragalus sinicus* L.)–rapeseed (*Brassica napus* L.) Intercropping.” Agriculture 12, no. 10: 1538. 10.3390/agriculture12101538.

[ece371149-bib-0032] Lu, S. , J. Lepo , H. X. Song , C. Y. Guan , and Z. H. Zhang . 2018. “Increased Rice Yield in Long‐Term Crop Rotation Regimes Through Improved Soil Structure, Rhizosphere Microbial Communities, and Nutrient Bioavailability in Paddy Soil.” Biology and Fertility of Soils 54, no. 4: 909–923. 10.1007/s00374-018-1315-4.

[ece371149-bib-0033] Lv, L. L. , H. L. Huang , J. T. Lv , et al. 2024. “Unique Dissolved Organic Matter Molecules and Microbial Communities in Rhizosphere of Three Typical Crop Soils and Their Significant Associations Based on FT‐ICR‐MS and High‐Throughput Sequencing Analysis.” Science of the Total Environment 919: 170904. 10.1016/j.scitotenv.2024.170904. 38354799

[ece371149-bib-0034] Morriën, E. , S. E. Hannula , L. B. Snoek , et al. 2017. “Soil Networks Become More Connected and Take Up More Carbon as Nature Restoration Progresses.” Nature Communications 8, no. 1: 14349. 10.1038/ncomms14349. PMC5309817 28176768

[ece371149-bib-0035] Morrissey, E. , J. Kane , B. Tripathi , et al. 2022. “Carbon Acquisition Ecological Strategies to Connect Soil Microbial Biodiversity and Carbon Cycling.” Soil Biology and Biochemistry 177: 108893. 10.1016/j.soilbio.2022.108893.

[ece371149-bib-0036] Muhammad Aslam, M. , E. Okal , A. Idris , et al. 2022. “Rhizosphere Microbiomes Can Regulate Plant Drought Tolerance.” Pedosphere 32: 61–74. 10.1016/S1002-0160(21)60061-9.

[ece371149-bib-0037] Olff, H. , and M. Ritchie . 1998. “Effects of Herbivores on Grassland Diversity.” Trends in Ecology & Evolution 13, no. 7: 261–265. 10.1016/S0169-5347(98)01364-0. 21238294

[ece371149-bib-0038] Peng, M. W. , H. He , M. Jiang , Z. K. Wang , G. F. Li , and L. Zhuang . 2023. “Morphological, Physiological and Metabolomic Analysis to Unravel the Adaptive Relationship Between Root Growth of Ephemeral Plants and Different Soil Habitats.” Plant Physiology and Biochemistry 202: 107986. 10.1016/j.plaphy.2023.107986. 37651954

[ece371149-bib-0039] Philippot, L. , J. Raaijmakers , P. Lemanceau , and W. Putten . 2013. “Going Back to the Roots: The Microbial Ecology of the Rhizosphere.” Nature Reviews. Microbiology 11: 789–799. 10.1038/nrmicro3109. 24056930

[ece371149-bib-0040] Sasse, J. , E. Martinoia , and T. Northen . 2017. “Feed Your Friends: Do Plant Exudates Shape the Root Microbiome?” Trends in Plant Science 23, no. 1: 25–41. 10.1016/j.tplants.2017.09.003. 29050989

[ece371149-bib-0041] Schimel, J. P. , J. M. Gulledge , J. S. Clein‐Curley , J. E. Lindstrom , and J. F. Braddock . 1999. “Moisture Effects on Microbial Activity and Community Structure in Decomposing Birch Litter in the Alaskan Taiga.” Soil Biology and Biochemistry 31, no. 6: 831–838. 10.1016/S0038-0717(98)00182-5.

[ece371149-bib-0042] She, J. Y. , J. Wang , X. D. Wei , et al. 2021. “Survival Strategies and Dominant Phylotypes of Maize‐Rhizosphere Microorganisms Under Metal (Loid)s Contamination.” Science of the Total Environment 774: 145143. 10.1016/j.scitotenv.2021.145143.

[ece371149-bib-0043] Shi, Y. H. , Y. S. Pan , L. Xiang , et al. 2021. “Assembly of Rhizosphere Microbial Communities in *Artemisia annua*: Recruitment of Plant Growth‐Promoting Microorganisms and Inter‐Kingdom Interactions Between Bacteria and Fungi.” Plant and Soil 470: 127–139. 10.1007/s11104-021-04829-9.

[ece371149-bib-0044] Siri‐Prieto, G. , M. Bustamante , V. Picasso , and O. Ernst . 2020. “Impact of Nitrogen and Phosphorous on Biomass Yield, Nitrogen Efficiency, and Nutrient Removal of Perennial Grasses for Bioenergy.” Biomass and Bioenergy 136: 105526. 10.1016/j.biombioe.2020.105526.

[ece371149-bib-0045] Solomon, W. , T. Janda , and Z. Molnár . 2024. “Unveiling the Significance of Rhizosphere: Implications for Plant Growth, Stress Response, and Sustainable Agriculture.” Plant Physiology and Biochemistry 206: 108290. 10.1016/j.plaphy.2023.108290. 38150841

[ece371149-bib-0046] Su, Y. W. , J. Wang , W. Y. Gao , et al. 2023. “Dynamic Metabolites: A Bridge Between Plants and Microbes.” Science of the Total Environment 899: 165612. 10.1016/j.scitotenv.2023.165612. 37478935

[ece371149-bib-0047] Tian, L. X. , Y. Feng , Z. J. Gao , et al. 2022. “Co‐Occurrence Pattern and Community Assembly of Broomcorn Millet Rhizosphere Microbiomes in a Typical Agricultural Ecosystem.” Applied Soil Ecology 176: 104478. 10.1016/j.apsoil.2022.104478.

[ece371149-bib-0048] Tohtahun, K. , D. Kong , L. Chai , et al. 2024. “Diversity and Growth‐Promoting Characteristics of Rhizosphere Bacteria of Three Naturally Growing Plants at the Sand Iron Ore Restoration Area in Qinghe County.” Science of the Total Environment 930: 172654. 10.1016/j.scitotenv.2024.172654. 38649044

[ece371149-bib-0049] Tong, Y. S. , Q. M. Dong , Y. Yu , et al. 2024. “Nitrogen Application Increases the Productivity of Perennial Alpine Cultivated Grassland by Improving Soil Physicochemical Properties and Microbial Community Characteristics.” Plant and Soil 505, no. 1‐2: 1–21. 10.1007/s11104-024-06694-8.

[ece371149-bib-0050] Tong, Y. Y. , X. Q. Zheng , Y. J. Hu , et al. 2024. “Root Exudate‐Mediated Plant–Microbiome Interactions Determine Plant Health During Disease Infection.” Agriculture, Ecosystems & Environment 370: 109056. 10.1016/j.agee.2024.109056.

[ece371149-bib-0051] Vives‐Peris, V. , C. D. Ollas , M. Aurelio.Gómez‐Cadenas , and R. Pérez‐Clemente . 2020. “Root Exudates: From Plant to Rhizosphere and Beyond.” Plant Cell Reports 39, no. 1: 3–17. 10.1007/s00299-019-02447-5. 31346716

[ece371149-bib-0052] Wang, J. , L. R. Liao , G. L. Wang , et al. 2022. “N‐Induced Root Exudates Mediate the Rhizosphere Fungal Assembly and Affect Species Coexistence.” Science of the Total Environment 804: 150148. 10.1016/j.scitotenv.2021.150148. 34520919

[ece371149-bib-0053] Wang, X. P. , M. Zhou , H. Yue , et al. 2024. “Effects of Different Artificial Vegetation Restoration Modes on Soil Microbial Community Structuree in the Soil Erosion Area of Southern China.” Catena 237: 107803. 10.1016/j.catena.2024.107803.

[ece371149-bib-0054] Wang, X. Y. , Y. Q. Li , X. W. Gong , et al. 2020. “Changes of Soil Organic Carbon Stocks From the 1980s to 2018 in Northern China's Agro‐Pastoral Ecotone.” Catena 194: 104722. 10.1016/j.catena.2020.104722.

[ece371149-bib-0055] Wang, Y. Z. , H. F. Zhang , Y. P. Zhang , et al. 2023. “Crop Rotation‐Driven Changes in Rhizosphere Metabolite Profiles Regulate Soil Microbial Diversity and Functional Capacity.” Agriculture, Ecosystems & Environment 358: 108716. 10.1016/j.agee.2023.108716.

[ece371149-bib-0056] Wani, A. K. , N. Akhtar , N. Naqash , et al. 2022. “Bioprospecting Culturable and Unculturable Microbial Consortia Through Metagenomics for Bioremediation.” Cleaner Chemical Engineering 2: 100017. 10.1016/j.clce.2022.100017.

[ece371149-bib-0057] Wani, A. K. , N. Akhtar , R. Singh , et al. 2022. “Prospects of Advanced Metagenomics and Meta‐Omics in the Investigation of Phytomicrobiome to Forecast Beneficial and Pathogenic Response.” Molecular Biology Reports 49: 12165–12179. 10.1007/s11033-022-07936-7. 36169892

[ece371149-bib-0058] Wani, A. K. , C. Chopra , M. A. Ansari , M. A. Dar , J. H. Américo‐Pinheiro , and R. Singh . 2024. “Characterization of Thermostable Carboxypeptidase From High‐Altitude Hot Spring Metagenome.” International Journal of Biological Macromolecules 276: 133974. 10.1016/j.ijbiomac.2024.133974. 39029824

[ece371149-bib-0059] Wani, A. K. , F. Rahayu , A. M. Alkahtani , et al. 2024. “Metagenomic Profiling of Rhizosphere Microbiota: Unraveling the Plant‐Soil Dynamics.” Physiological and Molecular Plant Pathology 133: 102381. 10.1016/j.pmpp.2024.102381.

[ece371149-bib-0060] Wei, W. , I. Kazuo , N. Tomoyasu , et al. 2015. “Higher Diversity and Abundance of Denitrifying Microorganisms in Environments Than Considered Previously.” ISME Journal 9, no. 9: 1954–1965. 10.1038/ismej.2015.9. 25756678 PMC4542046

[ece371149-bib-0061] Xu, C. G. , N. McDowell , F. Rosie , et al. 2019. “Increasing Impacts of Extreme Droughts on Vegetation Productivity Under Climate Change.” Nature Climate Change 9, no. 12: 948–953.

[ece371149-bib-0062] Yan, H. L. , S. S. Gu , S. Z. Li , et al. 2022. “Grass‐Legume Mixtures Enhance Forage Production via the Bacterial Community.” Agriculture, Ecosystems & Environment 338: 108087. 10.1016/j.agee.2022.108087.

[ece371149-bib-0063] Yang, L. , and S. Q. Zhao . 2023. “A Stronger Advance of Urban Spring Vegetation Phenology Narrows Vegetation Productivity Difference Between Urban Settings and Natural Environments.” Science of the Total Environment 868: 161649. 10.1016/j.scitotenv.2023.161649. 36657668

[ece371149-bib-0064] Yu, P. , X. M. He , M. Baer , et al. 2021. “Plant Flavones Enrich Rhizosphere Oxalobacteraceae to Improve Maize Performance Under Nitrogen Deprivation.” Nature Plants 7, no. 4: 481–499. 10.1038/s41477-021-00897-y. 33833418

[ece371149-bib-0065] Yuan, A. , S. D. Kumar , H. T. Wang , et al. 2024. “Dynamic Interplay Among Soil Nutrients, Rhizosphere Metabolites, and Microbes Shape Drought and Heat Stress Responses in Summer Maize.” Soil Biology and Biochemistry 191: 109357. 10.1016/j.soilbio.2024.109357.

[ece371149-bib-0066] Zhang, B. G. , J. Zhang , Y. Liu , P. Shi , and G. H. Wei . 2018. “Co‐Occurrence Patterns of Soybean Rhizosphere Microbiome at a Continental Scale.” Soil Biology and Biochemistry 118: 178–186. 10.1016/j.soilbio.2017.12.011.

[ece371149-bib-0067] Zhang, S. K. , J. Q. Kong , L. F. Chen , K. Guo , and X. D. Zhou . 2022. “Increased Tea Saponin Content Influences the Diversity and Function of Plantation Soil Microbiomes.” Microbiology Spectrum 10, no. 1: e0232421. 10.1128/spectrum.02324-21. 35019691 PMC8754145

[ece371149-bib-0068] Zhang, W. J. , X. Xue , F. Peng , Q. G. You , and A. H. Hao . 2019. “Meta‐Analysis of the Effects of Grassland Degradation on Plant and Soil Properties in the Alpine Meadows of the Qinghai‐Tibetan Plateau.” Global Ecology and Conservation 20: e00774. 10.1016/j.gecco.2019.e00774.

[ece371149-bib-0069] Zhao, M. L. , J. Zhao , J. Yuan , et al. 2021. “Root Exudates Drive Soil‐Microbe‐Nutrient Feedbacks in Response to Plant Growth.” Plant, Cell & Environment 44, no. 2: 613–628. 10.1111/pce.13928. 33103781

[ece371149-bib-0070] Zhao, N. , X. L. Zhang , L. Y. Hu , et al. 2022. “Cropping Practices Manipulate Soil Bacterial Structure and Functions on the Qinghai–Tibet Plateau.” Journal of Plant Physiology 271: 153666. 10.1016/j.jplph.2022.153666. 35303514

[ece371149-bib-0071] Zhao, Y. , Y. H. Yao , H. Y. Xu , et al. 2022. “Soil Metabolomics and Bacterial Functional Traits Revealed the Responses of Rhizosphere Soil Bacterial Community to Long‐Term Continuous Cropping of Tibetan Barley.” PeerJ 10: e13254. 10.7717/peerj.13254. 35415021 PMC8995024

[ece371149-bib-0072] Zhao, Z. Y. , Y. T. Ma , T. Y. Feng , et al. 2022. “Assembly Processes of Abundant and Rare Microbial Communities in Orchard Soil Under a Cover Crop at Different Periods.” Geoderma 406: 115543. 10.1016/j.geoderma.2021.115543.

[ece371149-bib-0073] Zhou, J. Q. , F. G. Zhang , Y. Q. Huo , et al. 2019. “Following Legume Establishment, Microbial and Chemical Associations Facilitate Improved Productivity in Degraded Grasslands.” Plant and Soil 443, no. 1‐2: 273–292. 10.1007/s11104-019-04169-9.

[ece371149-bib-0074] Zhuang, Y. , H. Wang , F. R. Tan , et al. 2024. “Rhizosphere Metabolic Cross‐Talk From Plant‐Soil‐Microbe Tapping Into Agricultural Sustainability: Current Advance and Perspectives.” Plant Physiology and Biochemistry 210: 108619. 10.1016/j.plaphy.2024.108619. 38604013

